# A noncanonical cytochrome *c* stimulates calcium binding by PilY1 for type IVa pili formation

**DOI:** 10.1073/pnas.2115061119

**Published:** 2022-02-04

**Authors:** Marco Herfurth, Anke Treuner-Lange, Timo Glatter, Nadine Wittmaack, Egbert Hoiczyk, Antonio J. Pierik, Lotte Søgaard-Andersen

**Affiliations:** ^a^Department of Ecophysiology, Max Planck Institute for Terrestrial Microbiology, 35043 Marburg, Germany;; ^b^Core Facility for Mass Spectrometry and Proteomics, Max Planck Institute for Terrestrial Microbiology, 35043 Marburg, Germany;; ^c^Biochemistry, Faculty of Chemistry, Technical University Kaiserslautern, D-67663 Kaiserslautern, Germany;; ^d^Department of Molecular Biology and Biotechnology, The Krebs Institute, The University of Sheffield, Sheffield S10 2TN, United Kingdom

**Keywords:** type IV pili, cytochrome *c*, PilY1, bacterial adhesin, minor pilin

## Abstract

Type IVa pili (T4aP) are versatile bacterial cell surface structures that undergo extension/adhesion/retraction cycles powered by the cell envelope–spanning T4aP machine. In this machine, a complex composed of four minor pilins and PilY1 primes T4aP extension and is also present at the pilus tip mediating adhesion. Similar to many several other bacteria, *Myxococcus xanthus* contains multiple minor pilins/PilY1 sets that are incompletely understood. Here, we report that minor pilins and PilY1 (PilY1.1) of cluster_1 form priming and tip complexes contingent on calcium and a noncanonical cytochrome *c* (TfcP) with an unusual His/Cys heme ligation. We provide evidence that TfcP is unlikely to participate in electron transport and instead stimulates calcium binding by PilY1.1 at low-calcium concentrations, thereby stabilizing PilY1.1 and enabling T4aP function in a broader range of calcium concentrations. These results not only identify a previously undescribed function of cytochromes *c* but also illustrate how incorporation of an accessory factor expands the environmental range under which the T4aP system functions.

## Significance

Type IVa pili (T4aP) are bacterial surface structures that function under different environmental conditions. In the machine for T4aP formation, a complex of minor pilins and PilY1 primes T4aP formation and is also present at the pilus tip mediating adhesion. Similar to several other bacterial adhesins, PilY1 depends on calcium binding for function. Here, we demonstrate that in *Myxococcus xanthus,* PilY1 at low levels of calcium depends on the accessory protein TfcP to bind calcium, thereby stabilizing the protein. TfcP is a noncanonical cytochrome *c* that does not participate in electron transport. Rather our data support that TfcP interacts transiently with PilY1 to stimulate calcium binding. In this way, TfcP expands the range of calcium levels under which T4aP functions.

In bacteria, motility is important for virulence, promotes colonization of habitats of diverse composition, and stimulates biofilm formation (1). Type IVa pili (T4aP) are filamentous cell surface structures that enable cell translocation across surfaces and also have critical functions in surface adhesion, surface sensing, host cell interaction, biofilm formation, predation, virulence, and DNA uptake (2–4). The versatility of T4aP is based on their ability to undergo cycles of extension, surface adhesion, and retraction (5, 6). Retractions generate a force up to 150 pN per pilus, pulling cells across surfaces (7).

**Fig. 1.**
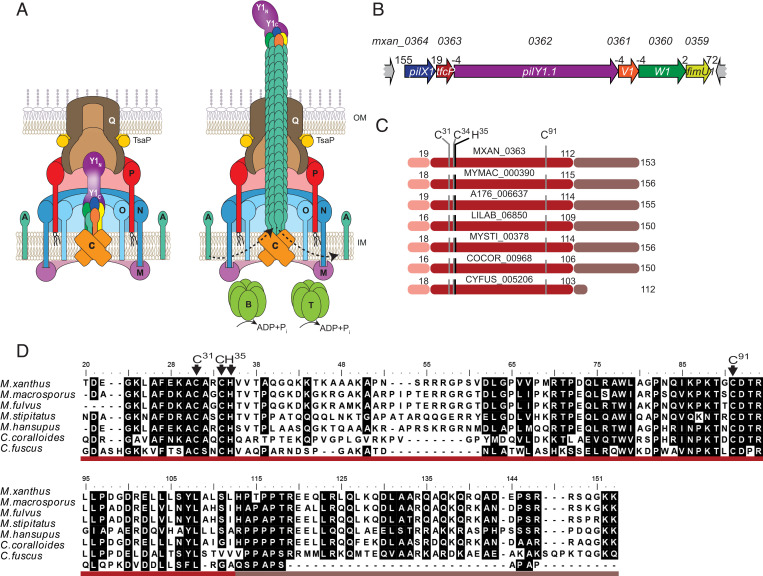
TfcP is a noncanonical cytochrome *c*. (*A*) Architectural model of nonpiliated and piliated T4aPM. PilB and PilT associate with PilC in a mutually exclusive manner during extension and retraction, respectively. Bent arrows, incorporation and removal of the major pilin PilA from the pilus base during extension and retraction, respectively. Proteins labeled with single letters have the Pil prefix. Y1_N_ and Y1_C_ indicate the N- and C-terminal domains of PilY1, respectively. The color code for the four minor pilins is as in *B*. (*B*) Genetic organization of cluster_1 encoding minor pilins, PilY1.1 and TfcP. Locus tags are included *Above* and gene names within genes. Distances between start and stop codons are shown *Above*. (*C*) Domain architecture of TfcP and homologs. Pink, type I signal peptide; red, cytochrome *c* domain; and brown, C-terminal extension. The cytochrome *c* signature motif CxxCH and the distal Cys^91^ residue are indicated. Numbering of amino acids is according to the unprocessed, full-length TfcP. (*D*) Sequence alignment of TfcP homologs. Residues are highlighted based on >80% similarity. Domains are indicated using the color code from *C*. The cytochrome *c* signature motif CxxCH and the distal Cys^91^ residue are indicated. Numbering of amino acids is according to the unprocessed, full-length proteins.

In Gram-negative bacteria, the extension/retraction cycles of T4aP are driven by the T4aP machine (T4aPM), which consists of 15 conserved proteins that form a complex that spans from the outer membrane (OM) across the periplasm and inner membrane (IM) to the cytoplasm (8–10) (Fig. 1*A*). Pilus extension and retraction are powered by the PilB and PilT ATPases, respectively, that bind in a mutually exclusive manner to the cytoplasmic base of the T4aPM (8, 11–13). All 15 proteins are essential for T4aP extension except for PilT, which is only important for retraction (4). The so-called priming complex is an integral part of the T4aPM, composed of the major pilin, four minor pilins and the PilY1 protein, and incorporated into the machine independently of the PilB ATPase (10, 14) (Fig. 1*A*). The five pilins interact directly to form a short pilus that is capped by PilY1, which interacts directly with the minor pilins (10). Pilus extension is initiated by the incorporation of additional major pilin subunits from a reservoir in the IM to the base of the priming complex in a process stimulated by PilB (6, 10, 14). Conversely, during retractions, major pilin subunits are removed from the base of the pilus and reinserted into the IM in a process stimulated by PilT (12, 15). Because the major pilin is added to the priming complex during the initiation of the extension process, the priming complex remains at the tip of the extended pilus (10, 14, 16). Consistently, PilY1 is involved in surface adhesion, surface sensing, specificity in host cell recognition during infections, and virulence (14, 16–19).

Among the 15 proteins of the T4aPM, 9 are generally encoded by single-copy genes (20). Some species contain multiple PilT paralogs that enable retractions with different characteristics (21). The genes for the four minor pilins and PilY1 are also often present in multiple copies (10, 22–24). The multiplicity of minor pilins and PilY1 proteins has been suggested to allow individual species to assemble priming complexes and tip complexes of different composition and with different properties, thereby allowing the formation of T4aP that can function in a variety of different habitats (10, 14, 25). Minor pilins are low-abundance proteins that share overall structure and sequence homology with the major pilin and have a prepilin signal peptide, a hydrophobic N-terminal α-helix, and a C-terminal globular domain, which is less conserved (26). PilY1 proteins have a type I signal peptide, are secreted to the periplasm, and are composed of two domains. The conserved C-terminal PilY1 domain adopts a beta-propeller fold (27) that interacts with the minor pilins in the priming and tip complex (10) (Fig. 1*A*). The N-terminal domain is much less conserved and is the domain that mediates host cell recognition, adhesion, and surface sensing (10, 17, 28).

The soil-dwelling δ-proteobacterium *Myxococcus xanthus* uses T4aP-dependent motility (T4aPdM) and gliding motility to move on surfaces to generate spreading colonies in the presence of nutrients and spore-filled fruiting bodies in the absence of nutrients (29, 30). The *M. xanthus* genome contains three gene clusters (from here on cluster_1, _2, and _3; proteins labeled with suffixes 1, 2, and 3), each encoding four minor pilins and a PilY1 protein (8, 10). Cluster_1 alone and cluster_3 alone support T4aPdM under standard conditions (10). While the four respective minor pilins share overall sequence homology, the three PilY1 proteins are highly divergent in their N-terminal domains (10). Thus, *M. xanthus* has the potential to generate at least two, and possibly three, different T4aPM and T4aP that differ in their priming and tip complexes.

To understand the functional range of the three minor pilin/PilY1 protein sets, we focused on the proteins of cluster_1. Here, we provide evidence that these proteins form priming and tip complexes in a calcium-dependent manner. We identify the TfcP protein and show that it is a noncanonical cytochrome *c* with an unusual His/Cys heme ligation that is important for PilY1.1 stability under low-calcium conditions; PilY1.1, in turn, is important for the stability of the cluster_1 minor pilins. The effect of TfcP on PilY1.1 stability depends on calcium binding by PilY1.1 and is bypassed at high-calcium concentrations. Our data support a model whereby TfcP promotes calcium binding by PilY1.1 at low-calcium concentrations, thereby, allowing cluster_1 to support T4aP function in a broader range of environmental conditions.

## Results

### TfcP Is a Noncanonical Cytochrome *C* Important for Cluster_1-Based T4aP Formation.

In addition to encoding four minor pilins (PilX1, PilW1, PilV1, and FimU1) and PilY1.1, cluster_1 contains an open reading frame (ORF) (locus tag=*mxan_0363*) (Fig. 1*B*), for which no homolog is present in cluster_2 and cluster_3. This ORF is conserved in gene clusters encoding minor pilins and PilY1 in other Myxococcales genomes (*SI Appendix*, Fig. S1*A*). Mxan_0363 homologs contain three parts, including a type I signal peptide (Fig. 1*C*). The middle part has similarity to cytochromes *c*, including a single cytochrome *c* signature motif CxxCH (31) (Fig. 1 *C* and *D* and *SI Appendix*, Fig. S1*B*). *C*-type cytochromes are secreted to the periplasm in a Sec-dependent manner where they acquire the heme, which is covalently attached to the two Cys residues in the signature motif by thioether bonds, while the His residue is the proximal axial ligand of the heme iron (32). Approximately 90% of cytochromes *c*, the so-called canonical cytochromes *c*, have a Met or His residue ∼60 residues downstream of the signature motif that serves as the second axial ligand of the heme iron (31, 33, 34) (*SI Appendix*, Fig. S1*B*). Interestingly, in Mxan_0363 and homologs, this is a Cys residue (Cys^91^ in Mxan_0363) (Fig. 1 *C* and *D* and *SI Appendix*, Fig. S1*B*) that is rarely found as the second axial ligand in *c*-type cytochromes (34, 35). In the vicinity of Cys^91^, no conserved Met or His residues are present and the alignment with canonical cytochromes *c* shows that Cys^91^ occupies the position of the conserved methionine distal ligand (*SI Appendix*, Fig. S1*B*). In an AlphaFold2 model together with ligand prediction using COACH (*SI Appendix*, *SI Materials and Methods*), this part of Mxan_0363 adopts a cytochrome *c*-like fold (36) that can readily be superimposed on the determined 1.5-Å structure (PDB 2B4Z) of *Bos taurus* cytochrome *c* (*SI Appendix*, Fig. S1*C*) and contains a heme c with His/Cys coordination (*SI Appendix*, Fig. S1*C*). Finally, Mxan_0363 and most of its homologs contain a C-terminal extension enriched in charged residues (Fig. 1 *C* and *D*), which does not occur in canonical cytochromes *c* (*SI Appendix*, Fig. S1 *B* and *C*). In the AlphaFold2 model, this extension is modeled as a 30-residue highly charged α-helix that is separated from the cytochrome *c* domain by a short Pro-rich region (*SI Appendix*, Fig. S1 *B* and *C*). Thus, Mxan_0363 homologs have features in common with canonical cytochromes *c* but also distinct features. Mxan_0363 homologs were not identified in species other than the listed Myxococcales (Fig. 1 *C* and *D*). From here on, we refer to Mxan_0363 as TfcP for T4aP formation cytochrome *c* protein.

Consistent with the overlap of or short distances between stop and start codons for neighboring genes in cluster_1 (Fig. 1*B*), they constitute an operon based on RT-PCR analyses (*SI Appendix*, Fig. S1*D*).

To test whether TfcP is important for T4aP formation or function, we generated in-frame deletions of *tfcP* and the remaining five cluster_1 genes. The deletions were generated in a strain in which cluster_2 and cluster_3 had been deleted (Δ2Δ3_cluster strain) because cluster_1 and _3 in the wild-type (WT) strain DK1622 function redundantly to support T4aP formation and T4aPdM (10). From here on, we used the Δ2Δ3_cluster strain as a reference strain and refer to it as the WT_Δ2Δ3_ strain.

**Fig. 2.**
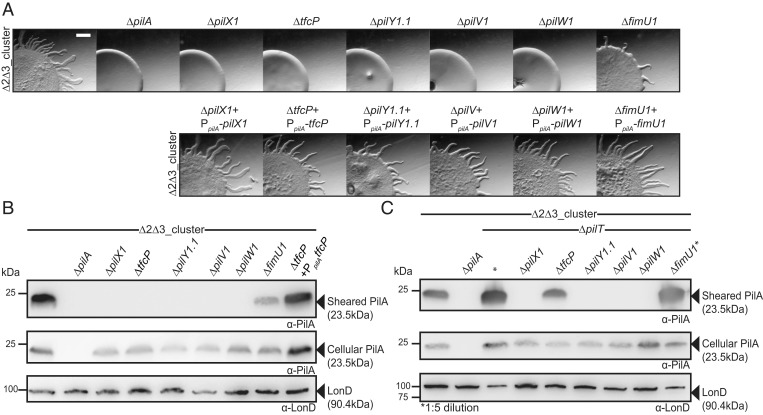
TfcP, minor pilins, and PilY1.1 of cluster_1 are important for T4aPdM and T4aP formation. (*A*) Assay for T4aPdM. WT_Δ2Δ3_ and strains with deletions of individual cluster_1 genes, and the corresponding complementation strains were spotted on 0.5% agar supplemented with 0.5% CTT and imaged after 24 h. (Scale bar, 1 mm.) (*B*) Shearing assay for T4aP formation. (*Top*) T4aP sheared off from ∼15 mg cells grown on 1.5% agar supplemented with 1.0% CTT were separated by sodium dodecyl sulphate-polyacrylamide gel electrophoresis (SDS-PAGE) and probed with α-PilA antibodies. (*Middle*) A total of 40 µg of total protein from total cell extracts separated by SDS-PAGE and probed with α-PilA antibodies and (*Lower*) after stripping, with α-LonD antibodies as a loading control. (*C*) Shearing assay for T4aP formation in retraction-deficient strains. T4aP formation was assayed as in *B*. In lanes labeled *, fivefold less total protein was loaded.

In motility assays for T4aPdM on 0.5% agar supplemented with 0.5% casitone broth (CTT), WT_Δ2Δ3_ generated the flares at the colony edge characteristic of T4aPdM, while the Δ*pilA* mutant, which lacks the major pilin PilA, did not (Fig. 2*A*). As for cluster_3 genes (10), T4aPdM was abolished in the Δ*pilX1*, Δ*pilV1*, Δ*pilW1*, and Δ*pilY1.1* mutants and reduced in the Δ*fimU1* mutant. Strikingly, T4aPdM was also abolished in the Δ*tfcP* mutant. For all six in-frame deletion mutants, motility was complemented by ectopic expression of the relevant gene from a plasmid integrated in a single copy at the Mx8 *attB* site (Fig. 2 *A*, *Lower*).

To pinpoint the mechanism causing the T4aPdM defect in the cluster_1 mutants, we assessed T4aP formation in the six in-frame deletion mutants using an assay in which T4aP are sheared off the cell surface, and the level of the major pilin PilA in the sheared fraction quantified by immunoblot analysis (Fig. 2*B*). None of the five nonmotile mutants formed detectable T4aP, while the Δ*fimU1* mutant assembled T4aP at a much-reduced level compared to the parent strain. For all six in-frame deletion mutants, the total cellular level of PilA was similar or slightly lower than in the parent WT_Δ2Δ3_ strain. T4aP formation in the Δ*tfcP* mutant was complemented by ectopic expression of *tfcP.*

To distinguish whether the defect in T4aP formation was caused by lack of extension or by hyperretractions, we examined T4aP formation in the in-frame deletion mutants additionally containing a Δ*pilT* mutation and, thus, lacking the PilT retraction ATPase (Fig. 2*C*). The WT_Δ2Δ3_Δ*pilT* strain formed T4aP at a highly increased level compared to WT_Δ2Δ3_, consistent with previous observations for the WTΔ*pilT* strain (12). In the absence of PilT, T4aP formation was partially restored in the Δ*tfcP* mutant, but at a much-reduced level compared to the WT_Δ2Δ3_Δ*pilT* strain. By contrast, T4aP formation in the Δ*pilX1*, Δ*pilV1*, Δ*pilW1*, and Δ*pilY1.1* mutants was not restored. For all in-frame deletion mutants except for the Δ*fimU1* mutant, the total cellular level of PilA was lower than in the WT_Δ2Δ3_Δ*pilT* strain. We conclude that TfcP is important but not essential for cluster_1-dependent T4aP extension while the minor pilins PilX1, -V1, and -W1 as well as PilY1.1 are essential for T4aP formation, and FimU1 plays a less important role. The observations are in agreement with similar experiments involving minor pilins and PilY1.3 of cluster_3 (10).

### TfcP Is Important for PilY1.1 Stability.

**Fig. 3.**
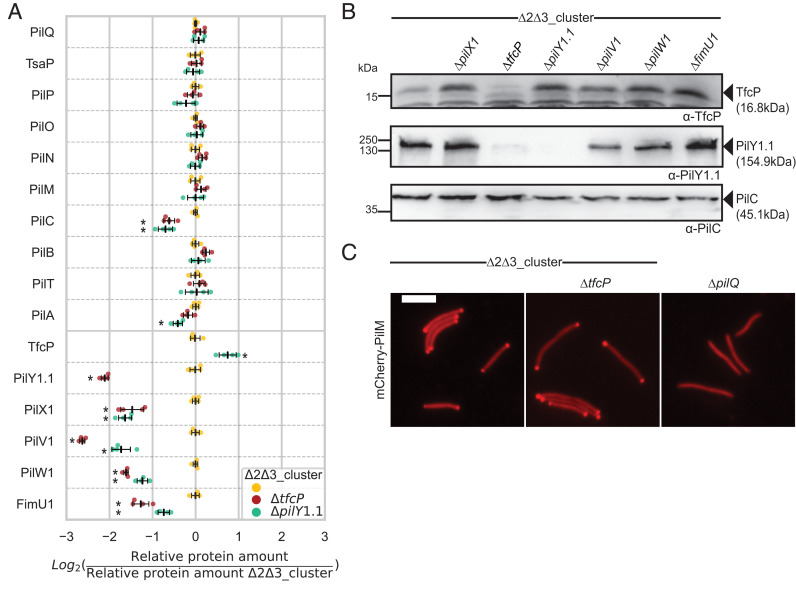
TfcP is important for stability of PilY1.1 and minor pilins of cluster_1. (*A*) Accumulation of proteins of the T4aPM and cluster_1. Cells were grown in 1.0% CTT suspension culture. Relative protein amounts were determined using targeted proteomics with one to five heavy-labeled reference peptides for each protein spiked into the trypsin-digested cell lysates (*SI Appendix*, *SI Materials and Methods*). To calculate relative protein amounts, each light-to-heavy intensity ratio of the endogenous (light) and reference (heavy) peptide was calculated. Individual data points represent the mean of the log_2_ ratios of the relative amount of all peptides of one protein in one biological replicate to the mean relative amount of the same peptide in the WT_Δ2Δ3_ strain. Center marker and error bars in black: Mean and SD from four biological replicates. Statistical analyses were performed by comparing WT_Δ2Δ3_ to the mutants using Welch’s test, *P < 0.01. (*B*) Immunoblot analysis of TfcP and PilY1.1 accumulation. Cells were grown in 1.0% CTT suspension culture. Total cell extracts from the same number of cells were separated by SDS-PAGE and analyzed by immunoblotting with the indicated antibodies. PilC was used as loading control. (*C*) Localization of mCherry-PilM. Strains were grown in 1.0% CTT suspension culture, placed on 1.0% agarose supplemented with Tris/phosphate/magnesium (TPM) buffer, and immediately imaged by fluorescence microscopy. (Scale bar, 5 µm.)

To understand how TfcP might be involved in T4aP extension, we used proteomics on whole-cell extracts to quantify the accumulation of T4aPM components in WT_Δ2Δ3_ and WT_Δ2Δ3_Δ*tfcP* strains. To increase sensitivity, we used targeted proteomics in which protein abundance is quantified relative to heavy-labeled reference peptides of the proteins of interest (*SI Appendix*, *SI Materials and Methods*). In the absence of TfcP, accumulation of 10 T4aPM components was largely unaffected, while the accumulation of the four minor pilins and PilY1.1 was significantly reduced (Fig. 3*A*). Because PilY1 of cluster_3 is important for the stability of cluster_3 minor pilins (10), we performed targeted proteomics on the WT_Δ2Δ3_Δ*pilY1.1* strain. In this strain, accumulation of the four minor pilins was also significantly reduced, while TfcP accumulation was significantly increased (Fig. 3*A*). In immunoblot analysis, we observed that in the absence of individual cluster_1 minor pilins, accumulation of TfcP was increased and PilY1.1 unchanged (Fig. 3*B*). Immunoblot analysis also confirmed that PilY1.1 accumulation was strongly reduced in the absence of TfcP, while TfcP accumulation was increased in the absence of PilY1.1 (Fig. 3*B*).

To resolve whether the effect of the Δ*tfcP* mutation on PilY1.1 and the Δ*pilY1.1* mutation on minor pilin accumulation was due to altered transcription of the relevant genes or altered protein stability, we performed qRT-PCR analysis on total RNA from the WT_Δ2Δ3_, WT_Δ2Δ3_Δ*tfcP*, and WT_Δ2Δ3_Δ*pilY1.1* strains. Transcript levels of the cluster_1 genes were significantly increased in the Δ*tfcP* and the Δ*pilY1.1* mutants (*SI Appendix*, Fig. S2), suggesting negative feedback regulation of cluster_1 genes as reported for the minor pilin/*pilY1* gene cluster of *Pseudomonas aeruginosa* (37). While the mechanism involved in this regulation remains unresolved, these results do not support the reduced levels of PilY1.1/minor pilins and minor pilins in the absence of TfcP and PilY1.1, respectively, being caused by reduced synthesis. Rather they support TfcP stabilizing PilY.1.1, which, in turn, stabilizes the four minor pilins. Accumulation dependencies have also been reported for the cluster_3 proteins in which PilY1.3 and minor pilins interact directly to mutually stabilize each other (10).

In *M. xanthus*, the T4aPM assembles at the two poles (10, 38–40). To exclude that lack of TfcP affects assembly of the T4aPM, we used the bipolar localization of the cytoplasmic protein PilM as proxy for T4aPM assembly (39). We observed bipolar localization of an active mCherry-PilM fusion in most cells of the WT_Δ2Δ3_ and WT_Δ2Δ3_Δ*tfcP* strains but not in a mutant lacking the PilQ secretin, which is essential for T4aPM assembly (39) (Fig. 3*C*), supporting the idea that TfcP is not important for assembly of the remaining proteins into rudimentary T4aPM.

### TfcP Is a Periplasmic Protein.

**Fig. 4.**
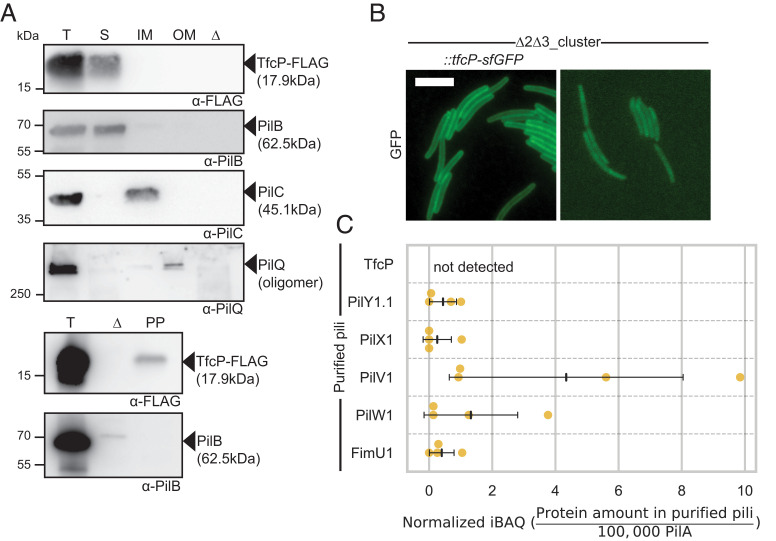
TfcP is a periplasmic protein. (*A*) Subcellular localization of TfcP-FLAG. Cells were grown in 1.0% CTT suspension culture and fractionated into fractions enriched for soluble proteins (S), IM proteins, and OM proteins (four *Upper* panels) or the periplasmic fraction (PP) (two *Lower* panels). T indicates total cell extract. In the lanes marked Δ, total cell extract of the Δ*pilBTCMNOPQ* mutant was used as negative control. Protein from the same number of cells was loaded per lane and analyzed by immunoblotting. PilB, PilC, and PilQ serve as controls for the fractionation and localize to the cytoplasm, IM and OM, respectively (12, 38). For PilQ, only the heat- and detergent-resistant oligomeric form is shown (39). (*B*) Determination of TfcP-sfGFP localization. Cells were grown in 1.0% CTT suspension culture, and analyzed as in Fig. 3*C*. WT_Δ2Δ3_ autofluorescence is shown as negative control. (Scale bar, 5 µm.) (*C*) Label-free quantification (LFQ) of cluster_1 proteins in purified pili. Pili were isolated from cells grown on 1.5% agar supplemented with 1.0% CTT. Normalized iBAQ (intensity based absolute quantification) values (*SI Appendix*, *SI Materials and Methods*) were determined in four biological replicates for WT_Δ2Δ3_Δ*pilT* and the negative control WT_Δ2Δ3_Δ*pilT*Δ*pilB*. iBAQ values of WT_Δ2Δ3_Δ*pilT* were background corrected by subtraction of the mean iBAQ value of the four replicates of the negative control and rescaled to the iBAQ value of 100,000 PilA molecules in the same sample. Center marker and error bars: Mean and SD.

To understand how TfcP stabilizes PilY1.1, we determined its subcellular localization using active TfcP-FLAG and TfcP-sfGFP fusions expressed from the endogenous locus; TfcP-FLAG accumulated at native levels while TfcP-sfGFP accumulated above native levels (*SI Appendix*, Fig. S3 *A* and *B*). After fractionation of WT_Δ2Δ3_ synthesizing TfcP-FLAG into fractions enriched for soluble, IM and OM proteins, TfcP-FLAG was detected in the soluble fraction while the control proteins fractionated as described (12, 38) (Fig. 4*A*). After isolating proteins enriched in the periplasm, we detected TfcP-FLAG but not cytoplasmic PilB (Fig. 4*A*). In agreement with these observations, in fluorescence microscopy, TfcP-sfGFP localized along the entire cell periphery but polar clusters were not observed (Fig. 4*B*). Based on these observations and because TfcP has a type I signal peptide, we conclude that TfcP, similarly to other cytochromes *c* in Gram-negative bacteria and PilY1 proteins (10), is a periplasmic protein. Because all proteins that are incorporated into the T4aPM localize (bi)polarly (10, 38–40), the localization of TfcP-sfGFP also suggests that TfcP is not incorporated into the T4aPM.

To determine whether TfcP is present in pili, we purified pili from the WT_Δ2Δ3_Δ*pilT* mutant (*SI Appendix*, Fig. S4) and used label-free quantitative proteomics to quantify cluster_1 proteins. TfcP was not detected in purified pili, while all minor pilins/PilY1 of cluster_1 were detected in low amounts relative to the PilA major pilin (Fig. 4*C*) as described for cluster_3 proteins (10).

Altogether, these observations support the idea that the minor pilins and PilY1.1 of cluster_1 form a priming complex in the T4aPM for T4aP extension as well as a pilus tip complex. The observations that TfcP stabilizes PilY1.1 but TfcP is incorporated into neither the T4aPM nor T4aP suggest that the stabilizing effect of TfcP on PilY1.1 occurs in the periplasm and before PilY1.1 incorporation into the T4aPM.

### TfcP Is a Noncanonical Cytochrome *C* with a Low Redox Potential and Heme Binding Is Important for TfcP Stability In Vivo.

We overexpressed and purified MalE-TfcP from *Escherichia coli* to assess TfcP’s heme-binding and redox characteristics. In size exclusion chromatography (SEC), MalE-TfcP eluted in a symmetric peak as a protein with a size of ∼62 kDa supporting the idea that it is monomeric and adopts a stable conformation (*SI Appendix*, Fig. S5 *A* and *B*). MalE-TfcP exhibited a distinct red color, indicating that it binds heme (*SI Appendix*, Fig. S5*C*). Oxidized MalE-TfcP had strong peroxidase activity (*SI Appendix*, Fig. S5*C*) in agreement with heme-containing proteins having intrinsic peroxidase activity (41). Importantly, peroxidase activity was inhibited when MalE-TfcP was reduced by dithiothreitol (DTT), supporting this activity as resulting from oxidized heme bound to MalE-TfcP (42).

**Fig. 5.**
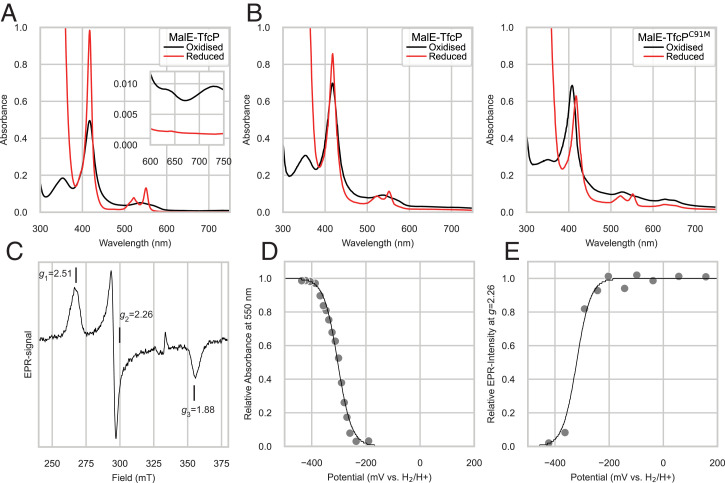
TfcP is a redox active, heme-binding protein. (*A*) UV-Vis spectra of purified MalE-TfcP in the oxidized and reduced (after addition of sodium-dithionite) state. *Inset*, absorbance in the 600- to 750-nm region. Experiment was done using a Shimadzu 1900 spectrophotometer. (*B*) UV-Vis spectra of purified MalE-TfcP variants in the oxidized and reduced state. Experiments were done using a Tecan200Pro plate reader and, therefore, the spectrum of WT TfcP is included again. (*C*) EPR spectrum of MalE-TfcP. Spectra were recorded in the oxidized state at 12 K, 0.32-mW microwave power, 1.5-mT modulation amplitude (9.3523 GHz). (*D*) Redox titration of MalE-TfcP. The 550-nm absorbance at 23 °C is plotted versus the solution potential and fitted to the Nernst equation. (*E*) Redox titration of MalE-TfcP following the EPR intensity at *g* = 2.26 of samples poised at indicated solution redox potentials.

To assess the heme-binding properties of TfcP, we used UV-visible (UV-Vis) spectroscopy. MalE-TfcP has a cytochrome *c*-like spectrum with a strong Soret peak in the oxidized form and after reduction with sodium-dithionite (Fig. 5*A*). In the spectrum of reduced TfcP, the α- and β-peaks become visible in the 550-nm region. This fits well to spectra of canonical cytochromes *c*. Interestingly, we did not observe a red shift of the Soret peak from the oxidized to the reduced spectrum. For canonical cytochromes *c* with His/His or His/Met coordination a 10-nm bathochromic shift is typically observed, while a semisynthetic cytochrome *c* with His/Cys coordination of the heme iron did not exhibit this shift (35, 43, 44), suggesting that Cys^91^ (Fig. 1 *C* and *D*) is the second axial ligand in TfcP and responsible for the lack of the red shift. This is also supported by the presence of a peak at 360 nm in the oxidized spectrum, which has been reported for His/Cys ligation (45). The presence of cysteine-to-Fe^3+^ charge transfer bands at ∼630 nm and ∼730 nm in the oxidized form, which disappear upon dithionite reduction (Fig. 5*A*, *Inset*), are also in full agreement with spectral properties of His/Cys coordinated *c*-type cytochromes (35). In control experiments, untagged TfcP also eluted from SEC as a monomeric protein (*SI Appendix*, Fig. S5 *D* and *E*) and was spectroscopically similar to MalE-TfcP (*SI Appendix*, Fig. S5 *F* and *G*). Therefore, to further support the special spectral properties of TfcP being due to Cys^91^, we purified MalE-TfcP^C91M^ (*SI Appendix*, Fig. S5*A*). In this variant, a red shift was observed upon reduction of the protein (Fig. 5*B*). In addition, the 360-nm peak was not detected in the oxidized form. We conclude that Cys^91^ is the second axial ligand of the heme iron in TfcP and that TfcP is a noncanonical cytochrome *c.*

We used electron paramagnetic resonance (EPR) spectroscopy to investigate the environment of the heme center. We obtained *g* values of 2.51, 2.26, and 1.88 (Fig. 5*C*) that fit well to the *g* values observed for multiple heme-containing proteins with a cysteine thiolate–ligated heme iron (46). Cytochromes *c* with a distal Cys were reported to have a very low midpoint potential in the range of −350 mV, while canonical cytochromes *c* have a potential of approximately +250 mV (35, 43, 44, 47). To determine whether TfcP has a similarly low redox potential, we used UV-Vis and EPR-monitored redox titrations (Fig. 5 *D* and *E*). For the UV-Vis redox titration, MalE-TfcP was incubated with a redox-mediator mixture. Spectra and potentials were recorded in 5-min intervals after addition of sodium-dithionite. After plotting the absorbance change at 550 nm versus the potential and fitting to the Nernst equation, the midpoint potential was determined as E_m_ = −304 ± 8 mV, where 8 mV represent the intrinsic fitting error in one experiment. In the EPR-monitored redox titration, we followed the change of *g* = 2.26 EPR signal in frozen samples obtained by sequential reduction with sodium-dithionite in the presence of mediators and found a midpoint potential of E_m_ = −320 ± 15 mV, where 15 mV represents the intrinsic fitting error in one experiment. Overall, both experiments support the idea that TfcP has a very low redox potential. The slight difference between the two experiments is likely due to pH changes, which occur during freezing. The low redox potential (approximately −312 mV) indicates that TfcP is not likely to be part of a respiratory chain in *M. xanthus* (*Discussion*). Supporting this notion, WT_Δ2Δ3_ and WT_Δ2Δ3_Δ*tfcP* had similar growth rates (*SI Appendix*, Fig. S6*A*).

To clarify whether the heme-binding characteristics of TfcP are important in vivo, we substituted the two Cys residues to Ala in the C^31^xxCH motif and Cys^91^ to His or Met (Fig. 1 *C* and *D*). The variants were synthesized ectopically as FLAG-tagged proteins in the Δ*tfcP* mutant from the strong *pilA* promoter. The three mutant variants accumulated at much-reduced levels compared to TfcP and TfcP-FLAG expressed from the native site, and supported neither PilY1.1 accumulation nor motility (*SI Appendix*, Fig. S6 *B* and *C*). We conclude that heme binding and distal coordination of the heme iron are important for TfcP stability.

A FLAG-tagged TfcP^Δ118–153^ variant lacking the C-terminal α-helical extension also accumulated at reduced levels and supported neither PilY1.1 accumulation nor motility (*SI Appendix*, Fig. S6 *B* and *C*), providing experimental support for the importance of this extension for protein stability.

### Added Calcium Restores T4aP Formation in the Absence of TfcP.

**Fig. 6.**
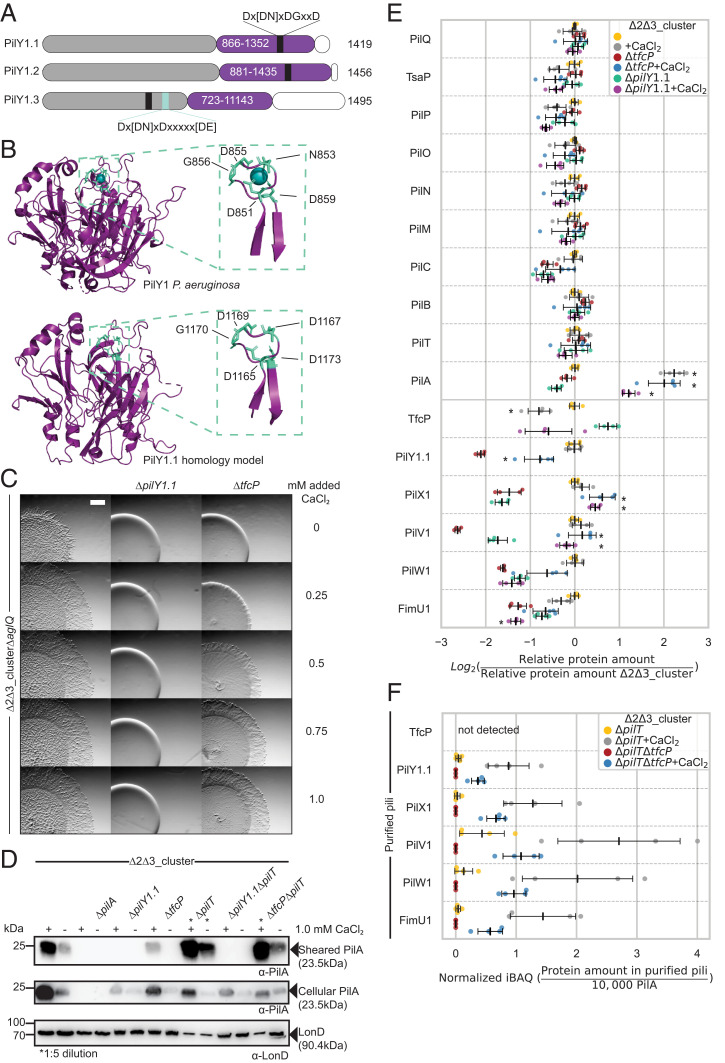
Added CaCl_2_ compensates for lack of TfcP. (*A*) Domain architecture of PilY1 proteins of *M. xanthus*. Purple, C-terminal PilY1 domain; gray, N-terminal domain; white; C-terminal sequences. EF-hand–like calcium binding motif is in black together with the consensus sequence; light blue box indicates second calcium binding motif in PilY1.3 together with the consensus sequence (25). (*B*) Comparison of PilY1 structure of *P. aeruginosa* (PDB 3HX6) (27) and a homology model of PilY1.1. *Inset*, zoom of calcium binding motif. (*C*) Assay for T4aPdM. Cells were grown in 1.0% CTT suspension culture and plated on 0.5% agar supplemented with 0.5% CTT and the indicated final concentrations of added CaCl_2_, and imaged after 24 h. Note that the flares formed by WT_Δ2Δ3_Δ*aglQ* are shorter than those formed by WT_Δ2Δ3_ due to the Δ*aglQ* mutation. (Scale bar, 1 mm.) (*D*) Shearing assay for T4aP formation. T4aP sheared off from ∼15 mg cells grown on 1.5% agar supplemented with 1.0% CTT and 1.0 mM CaCl_2_ as indicated, and analyzed as in Fig. 2*B*. (*E*) Accumulation of proteins of the T4aPM. Cells were grown in 1.0% CTT suspension culture without or with 1.0 mM added calcium as indicated. Proteins were quantified as in Fig. 3*A*. Data for samples without added CaCl_2_ are the same as in Fig. 3*A* and included for comparison. Statistical analyses were done by comparing cells grown in the presence versus the absence of calcium using Welch’s test, *P < 0.01. (*F*) LFQ proteomics of cluster_1 proteins in purified pili. Pili were isolated as in Fig. 4*C* after growth without or with added CaCl_2_ as indicated. Normalized iBAQ values were calculated as in Fig. 4*C* and background corrected by subtraction of the mean iBAQ value of the four replicates of the relevant negative control, and rescaled to 10,000 PilA molecules in the same sample. Data for WT_Δ2Δ3_ without added CaCl_2_ are the same as in Fig. 4*C* and included for comparison.

Several PilY1 proteins have been shown to bind calcium using an EF-hand–like motif in the C-terminal domain, and calcium binding is important for function (27, 48, 49). PilY1.1 and PilY1.2 contain the consensus EF-hand–like calcium binding Dx[DN]xDGxxD motif in the C-terminal PilY1 domain, while PilY1.3 has two calcium binding motifs in the N-terminal domain (Fig. 6*A*). In a homology model of the C-terminal domain of PilY1.1, the D^1165^xDxDNxxD^1173^ motif is located in a surface exposed loop between two β-strands as described for the *P. aeruginosa* PilY1 domain (27) (Fig. 6*B*).

To address the effect of calcium on T4aPdM, we considered that the previous experiments were performed either in 1.0% CTT (targeted proteome analyses and qRT-PCR), which has a calcium concentration of ∼30 µM according to the manufacturer, or on 0.5% agar supplemented with 0.5% CTT (motility assays) or 1.5% agar supplemented with 1.0% CTT (T4aP purification). The estimated calcium concentration of 0.5% agar is ∼0.15 mM (50). To assess the effect of added CaCl_2_ on T4aPdM, we used the WT_Δ2Δ3_Δ*aglQ* strain, which lacks the AglQ motor for gliding (51, 52). In the presence of ≥0.25 mM added CaCl_2_, WT_Δ2Δ3_Δ*aglQ* exhibited a dramatic change of motility pattern from expansion in flares to a radial film-like expansion (Fig. 6*C*). Intriguingly, the WT_Δ2Δ3_Δ*aglQ*Δ*tfcP* mutant also responded to external calcium, and at added CaCl_2_ concentrations ≥0.5 mM, this mutant regained T4aPdM and at 1.0 mM displayed a motility pattern similar to that of the parent strain. By contrast, added CaCl_2_ did not restore T4aPdM in the WT_Δ2Δ3_Δ*pilY1.1* mutant even at 10 mM (Fig. 6*C* and *SI Appendix*, Fig. S7 *A* and *B*). Likewise, 10 mM CaCl_2_ did not restore T4aPdM in the Δ*pilX1*, Δ*pilV1*, Δ*pilW1*, and Δ*pilA* mutants while the Δ*fimU1* mutant displayed the same radial motility pattern as the parent strain (*SI Appendix*, Fig. S7*A*). Additional experiments demonstrated that WT_Δ2Δ3_Δ*aglQ* responded to added CaCl_2_ concentrations as low as 0.025 mM while the WT_Δ2Δ3_Δ*aglQ*Δ*tfcP* only responded at ≥0.5 mM (*SI Appendix*, Fig. S7*B*). In control experiments, neither 5 mM NaCl nor 5 mM MgCl_2_ restored motility in the WT_Δ2Δ3_Δ*tfcP* mutant. A strain containing only cluster_3 responded to CaCl_2_ with an altered expansion pattern; however, this pattern was only evident at added CaCl_2_ concentrations ≥0.25 mM (*SI Appendix*, Fig. S7*B*). We conclude that CaCl_2_ at an added concentration of 1.0 mM restores T4aPdM in the WT_Δ2Δ3_Δ*tfcP* strain. From here on, we used an added CaCl_2_ concentration of 1.0 mM.

Consistent with the effect of CaCl_2_ on motility, WT_Δ2Δ3_Δ*tfcP* formed T4aP in the presence of 1.0 mM CaCl_2_, while the WT_Δ2Δ3_Δ*pilY1.1* mutant did not (Fig. 6*D*). Added CaCl_2_ also increased the amount of pili in WT_Δ2Δ3_. The level of T4aP in the WT_Δ2Δ3_Δ*tfcP* mutant was lower than in WT_Δ2Δ3_ (Fig. 6*D*). Calcium also increased the amount of total cellular PilA in all strains (Fig. 6*D*). The retraction-deficient strains WT_Δ2Δ3_Δ*pilT* and WT_Δ2Δ3_Δ*tfcP*Δ*pilT* assembled more T4aP in the presence of added CaCl_2_ than in its absence, supporting the idea that calcium stimulates T4aP formation rather than counteracting retractions (Fig. 6*D*). Thus, 1.0 mM of added CaCl_2_ can substitute for TfcP function in T4aP formation and T4aPdM.

### TfcP Enhances Calcium-Dependent Stabilization of PilY1.1.

To understand how a high concentration of calcium compensates for lack of TfcP, we used targeted proteomics. In WT_Δ2Δ3_, 1.0 mM of CaCl_2_ caused a significant increase in PilA abundance and a significant decrease in TfcP abundance, while accumulation of other T4aPM components including the remaining cluster_1 proteins was largely unaffected (Fig. 6*E*). In WT_Δ2Δ3_Δ*tfcP*, added CaCl_2_ not only caused a significant increase in PilA abundance but also significantly increased the abundance of most remaining cluster_1 proteins including PilY1.1 (Fig. 6*E*). In WT_Δ2Δ3_Δ*pilY1.1*, extra CaCl_2_ also caused increased PilA abundance, but a reduction in TfcP abundance as in the WT_Δ2Δ3_ parent strain. PilW1 and FimU1 abundance was unaffected by added CaCl_2_ in WT_Δ2Δ3_Δ*pilY1.1*, while PilV1 and PilX1 abundance significantly increased. We conclude that a high concentration of CaCl_2_ causes increased PilY1.1 accumulation in the absence of TfcP. Extra CaCl_2_ also caused 1) increased PilA accumulation independently of TfcP and PilY1.1, 2) decreased accumulation of TfcP independently of PilY1.1, and 3) increased accumulation of the minor pilins PilX1 and PilV1 independently of PilY1.1.

Because changes in extracellular calcium can cause altered gene expression (53), we performed qRT-PCR analyses to discriminate whether added CaCl_2_ affects transcription or protein stability. We observed significant changes in the transcription of all cluster_1 genes as well as of *pilA* in response to added CaCl_2_ (*SI Appendix*, Fig. S8); however, generally, these changes did not correlate with the altered protein accumulation profiles. For instance, in WT_Δ2Δ3_, 1.0 mM added CaCl_2_ caused increased PilA accumulation but *pilA* transcription was decreased, and decreased *pilY1.1* transcription but PilY1.1 abundance remained unchanged; and, in WT_Δ2Δ3_Δ*tfcP*, CaCl_2_ caused decreased *pilY1.1* transcription but PilY1.1 abundance increased. We conclude that added CaCl_2_ at 1.0 mM can substitute for TfcP in stabilizing PilY1.1.

Label-free quantitative proteomics of purified pili from WT_Δ2Δ3_Δ*pilT* and WT_Δ2Δ3_Δ*pilT*Δ*tfcP* (*SI Appendix*, Fig. S4) revealed a strong increase in the abundance of cluster_1 minor pilins and PilY1.1 relative to PilA in the presence of calcium (Fig. 6*F*), suggesting that calcium also stabilizes minor pilins and PilY1.1 in the tip complex. Of note, TfcP was not detected in purified pili from WT_Δ2Δ3_Δ*pilT* grown in the presence of added calcium (Fig. 6*F*).

To determine whether the effect of calcium on PilY1.1 stability depends on its binding to PilY1.1, we attempted to purify full-length PilY1.1 or its C-terminal domain but were unsuccessful, thus, precluding in vitro analyses of PilY1.1. Therefore, to assess calcium binding by PilY1.1 in vivo, we introduced the Asp^1173^ to Ala substitution in the EF-hand–like calcium binding D^1165^xDxDNxxD^1173^ motif in PilY1.1 (Fig. 6 *A* and *B*) and expressed the protein from the native site in WT_Δ2Δ3_ strains. The homologous substitution in other PilY1 proteins disrupts calcium binding without affecting the overall structure of the C-terminal beta-propeller domain (27, 48, 49).

**Fig. 7.**
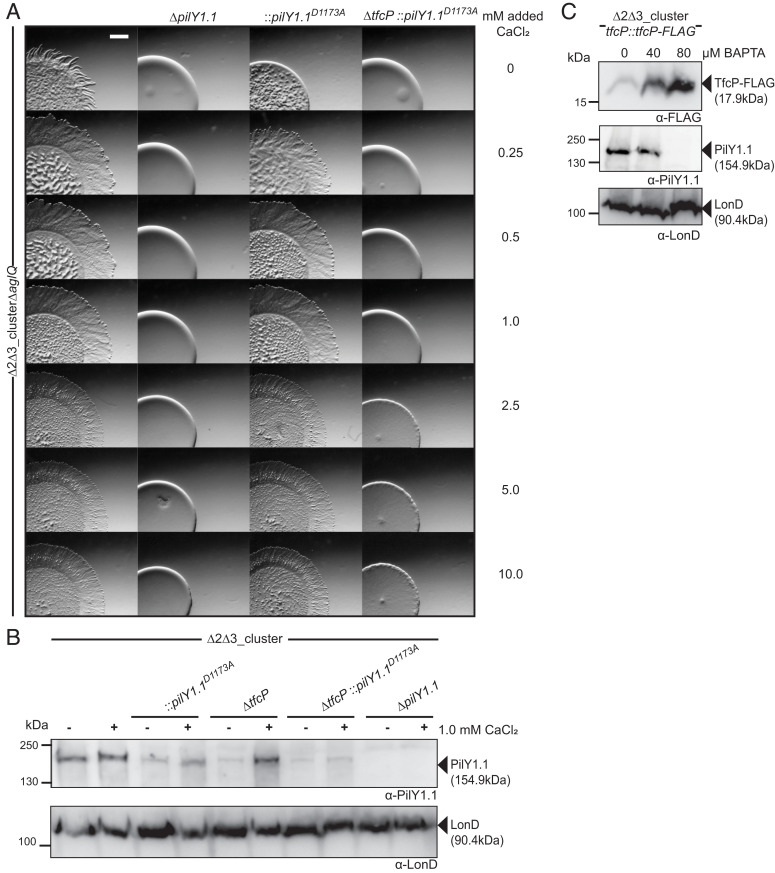
Calcium binding by PilY1.1 is essential for TfcP function. (*A*) Assay for T4aPdM. Cells were grown in 1.0% CTT suspension culture and plated on 0.5% agar supplemented with 0.5% CTT and imaged after 24 h. The final concentration of added CaCl_2_ is indicated. (Scale bar, 1 mm.) (*B*) Accumulation of PilY1.1 variants. Cells were grown in 1.0% CTT suspension culture without or with 1.0 mM CaCl_2_, total cell extract isolated and analyzed by immunoblot as in Fig. 3*B*. (*C*) Accumulation of TfcP-FLAG and PilY1.1 in the presence of BAPTA. Cells were grown in 1.0% CTT in suspension, exposed to indicated concentrations of BAPTA for 16 h, and total cell extract was isolated and analyzed by immunoblot as in Fig. 3*B*.

The *pilY1.1*^D1173A^*tfcP*^+^ mutant was strongly reduced in T4aPdM in the absence of added CaCl_2_ (Fig. 7*A*); however, this strain regained T4aPdM and was indistinguishable from the parent strain at ≥0.25 mM added CaCl_2_. By contrast, the *pilY1.1*^D1173A^Δ*tfcP* strain was nonmotile even at 10 mM of added CaCl_2_. These observations support the notion that calcium binding is important for PilY1.1 function and that PilY1.1^D1173A^ is fully functional at elevated calcium concentrations but only if TfcP is present.

Consistent with the observations for T4aPdM, PilY1.1^D1173A^ accumulation was reduced in the *pilY1.1*^D1173A^*tfcP*^+^ mutant in the absence of added CaCl_2_, and 1.0 of mM CaCl_2_ at least partially restored its accumulation (Fig. 7*B*). By contrast, in the *pilY1.1*^D1173A^Δ*tfcP* strain, PilY1.1^D1173A^ was detected at very low levels in the absence as well as in the presence of added CaCl_2_. Thus, PilY1.1^D1173A^ depends on TfcP for stability and responds to added calcium only in the presence of TfcP. By comparison, PilY1.1^WT^ is fully functional at ≥1.0 mM added CaCl_2_ in the absence of TfcP (Fig. 6*C*).

To determine whether TfcP can stabilize PilY1.1^WT^ independently of calcium, we analyzed PilY1.1 accumulation in the presence of the highly specific calcium chelator BAPTA (1,2-bis(*o*-aminophenoxy)ethane-N,N,N′,N′-tetraacetic acid). In WT_Δ2Δ3_ expressing TfcP-FLAG from the endogenous site and grown in 1.0% CTT, PilY1.1 was detected in the presence of 40 µM but not in the presence of 80 µM BAPTA, while TfcP was detected under all conditions and increased upon BAPTA addition (Fig. 7*C*). These observations strongly support the idea that TfcP can only stabilize PilY1.1 in the presence of calcium. Because CaCl_2_ can stabilize PilY1.1 in the absence of TfcP, these observations suggest that the primary function of TfcP is to stimulate calcium binding by PilY1.1 at low-calcium concentrations.

Finally, using a fluorescence-based assay, we observed that purified untagged TfcP did not detectably bind calcium (*SI Appendix*, Fig. S9). Consistently, TfcP does not contain a conserved calcium binding motif.

## Discussion

**Fig. 8.**
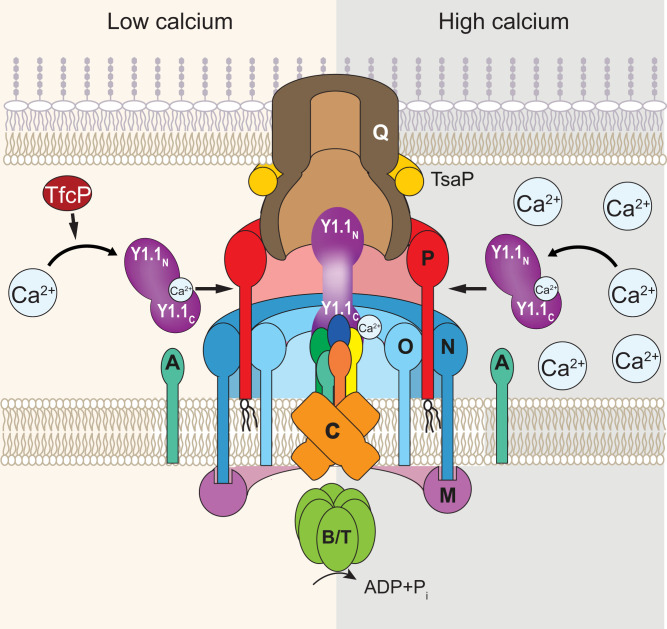
Model of TfcP function at low- and high-calcium concentrations. For simplicity, PilB and PilT are not indicated separately. Y1.1_N_ and Y1.1_C_ indicate the N- and C-terminal domains of PilY1, respectively. The color code for the four minor pilins is as in Fig. 1*B*.

Here, we identify TfcP, a noncanonical cytochrome *c*, as important for cluster_1-dependent T4aP formation in *M. xanthus* at low-calcium concentrations. We demonstrate that TfcP stabilizes PilY1.1 at low-calcium concentrations. PilY1.1, in turn, stabilizes the four minor pilins of cluster_1 in that way enabling the formation of the cluster_1-based priming complex in the T4aPM and, thus, T4aP formation. Bacteria in their natural habitats experience large fluctuations in environmental conditions and depend on adaptive strategies to endure such changes. TfcP expands the range of calcium concentrations under which cluster_1 encoded minor pilins and PilY1.1 can support T4aPdM, thereby increasing fitness of *M. xanthus* under changing environmental conditions and enabling colonization of habitats with low-calcium concentrations (Fig. 8).

Several lines of evidence support the idea that the effect of TfcP on PilY1.1 stability is calcium dependent. First, under standard conditions, *M. xanthus* is exposed to ∼30 µM calcium in CTT suspension culture and ∼0.15 mM calcium on 0.5% agar plates for motility assays. Under these conditions, TfcP is important for PilY1.1 stability. However, at concentrations ≥1 mM of added CaCl_2_, calcium alone is sufficient to stabilize PilY1.1 independently of TfcP. Second, in the complete absence of calcium, i.e., after addition of the calcium-specific chelator BAPTA, TfcP does not stabilize PilY1.1 while TfcP still accumulates. Third, the PilY1.1^D1173A^ variant, which is predicted to bind calcium with reduced affinity, depends on TfcP for stability at 1.0 mM added CaCl_2_, and even at 10 mM CaCl_2_, this protein is nonfunctional in the absence of TfcP. Thus, TfcP and calcium both function to stabilize PilY1.1. However, while high-calcium concentrations alone can stabilize PilY1.1, TfcP cannot stabilize PilY1.1 in the absence of calcium. Altogether, these findings support a model whereby calcium binding by PilY1.1 is the primary determinant for its stability and in which TfcP stabilizes PilY1.1 at low-calcium concentrations by stimulating calcium binding by PilY1.1. The functional outcome of this stimulation is that PilY1.1 accumulates at low-calcium concentrations and is able to support cluster_1-dependent T4aP formation and T4aPdM. Many myxobacteria, including *M. xanthus*, are found in terrestrial habitats in which calcium concentrations are described to vary from 0.1 to 1.0 mM at root–soil interfaces, 3.4 to 14 mM in some soils, and as low as 10 to 150 µM in other soils (54). Because the OM has been reported to be permeable to calcium, the environmental calcium concentration directly affects the periplasmic calcium concentration (55). We suggest that TfcP is key to enabling PilY1.1-dependent T4aP formation and T4aPdM in the lower range of environmental calcium concentrations. Interestingly, *Myxococcus stipitatus* and *Corallococcus coralloides* have only one gene cluster for minor pilins and PilY1, and this cluster encodes a TfcP ortholog (Fig. 1*C* and *SI Appendix*, Fig. S1*A*) emphasizing the importance of TfcP in T4aPdM in myxobacteria.

TfcP is a soluble, periplasmic protein and contains a noncanonical cytochrome *c* domain in which the second axial heme ligand is a Cys residue rather than the more common His and Met residues in canonical cytochromes *c*. Accordingly, TfcP has a very low redox potential of −304 to −320 mV based on two methods. Moreover, TfcP variants unable to bind heme or with altered heme-binding properties are unstable in vivo. *M. xanthus* is strictly aerobic and the genome encodes complex I through IV of the electron transport chain (56). Thus, the low redox potential of TfcP supports the idea that it is not part of the respiratory chain, which starts with a potential of −320 mV for the redox pair NAD/NADH (57). Some cytochromes *c* are involved in electron transport across the OM to external electron acceptors; however, these proteins are canonical cytochromes *c* (58), suggesting that TfcP also does not engage in this type of electron transport. Some *c*-type cytochromes with His/Cys ligation, e.g., the triheme DsrJ of *Allochromatium vinosum*, are involved in dissimilatory sulfur metabolism in which sulfate is used as the terminal electron acceptor (59), and some have been suggested to have a role in signaling (35, 60). Because *M. xanthus* does not respire on sulfate, it is unlikely that TfcP would be involved in dissimilatory sulfur metabolism. While we cannot rule out a function of TfcP in signaling, our data support a scenario in which TfcP is a repurposed cytochrome *c* that is no longer involved in electron transport, and in which the covalently bound heme serves a structural function to stabilize TfcP. This “inert” cytochrome *c* then stimulates calcium binding by PilY1.1 at low-calcium concentrations. TfcP also differs from canonical cytochromes *c* by having a highly charged C-terminal extension. This extension is important for TfcP stability; however, its precise function remains to be uncovered.

An Asp to Ala substitution in the calcium binding motif in the PilY1 proteins of *P. aeruginosa*, *Kingella kingae*, and *Neisseria gonorrhoeae* abolishes calcium binding and renders the proteins nonfunctional while still folding correctly and accumulating (27, 48, 49). The corresponding PilY1.1^D1173A^ variant was also functionally impaired, supporting the idea that PilY1.1 binds calcium as described for other PilY1 proteins. Compared to PilY1.1^WT^, PilY1.1^D1173A^ had an increased dependency on TfcP and calcium for stability, indicating that PilY1.1 depends more strongly on calcium binding for stability than other PilY1 proteins. More importantly, PilY1.1^D1173A^ was still functional at high-calcium concentrations but only in the presence of TfcP. Thus, TfcP can rescue the PilY1.1^D1173A^ calcium-binding mutant, emphasizing the role of TfcP in stimulating calcium binding by PilY1.1.

The observation that PilY1.1 is unstable in the absence of TfcP at low-calcium concentrations suggest that the two proteins interact directly. However, such an interaction remains to be shown and will be addressed in future experiments. Nevertheless, some inferences can be made. PilY1.1 and the four minor pilins of cluster_1 were detected in purified pili. By contrast, TfcP was not detected in purified pili. We previously showed that sfGFP-tagged PilY1.3 and the sfGFP-tagged minor pilin PilW3 of cluster_3 localize polarly, are incorporated into the T4aPM but do not support pilus extension, likely because the sfGFP-tag jams the machine by precluding passage of PilY1.3-sfGFP and PilW3-sfGFP through the secretin channel in the OM (10). By contrast, TfcP-sfGFP was fully active and did not localize polarly. These observations strongly support TfcP as neither part of the pilus nor the T4aPM. They also strengthen the hypothesis that the suggested direct interaction between PilY1.1 and TfcP is transient and only occurs in the periplasm before PilY1.1 incorporation into the T4aPM (Fig. 8). The observations that added calcium stabilizes PilY1.1 in the absence of TfcP and that TfcP does not bind calcium supports TfcP as not acting as a metallochaperone to deliver calcium to PilY1.1. We, therefore, suggest that TfcP interacts transiently with calcium-free PilY1.1 prior to PilY1.1 incorporation into the T4aPM. We suggest that TfcP either supports correct PilY1.1 folding or induces conformational changes in PilY1.1, thereby enabling efficient calcium binding by PilY1.1. Subsequently, calcium-bound PilY1.1, but not TfcP, is incorporated into the priming complex of the T4aPM to support T4aP formation (Fig. 8). This mechanism of protein stabilization is reminiscent of that of periplasmic chaperones, which in an ATP-independent manner transiently interact with their periplasmic clients to enable folding (61), except that the suggested TfcP/PilY1.1 interaction promotes calcium binding by PilY1.1, which then stabilizes PilY1.1. Altogether, these findings also provide evidence for a previously undescribed function of a cytochrome *c* in protein folding and/or stabilization.

In addition to the conserved proteins of the T4aPM, T4aP extension in several species depends on accessory factors that are much less conserved. For instance, the c-di-GMP binding protein FimX in *P. aeruginosa* and SgmX in *M. xanthus* stimulate T4aP extension (62–64). TfcP adds to the list of such regulators and also acts at the level of extension; however, in contrast to these cytoplasmic regulators, TfcP acts in the periplasm.

In the presence of added CaCl_2_ at 1.0 mM, more T4aP are formed and the ratio between minor pilins and PilY1.1 to PilA is increased. These observations support calcium as not only helping to stabilize PilY1.1, but may also stabilize the pilus, including the minor pilin/PilY1.1 tip complex in extracellular space. In this context, it is interesting to note that calcium binding has been reported to stabilize the interactions between major pseudopilin subunits in the pseudopilus of the type II secretion system of *Klebsiella oxytoca* (65). In future experiments, this effect of calcium will be addressed.

## Materials and Methods

### Bacterial Strains and Growth Media.

All *M. xanthus* strains are derivatives of DK1622 (66) and listed in *SI Appendix*, Table 1. Plasmids are listed in *SI Appendix*, Table 2. In-frame deletion mutants were generated using double homologous recombination using a *galK-*containing plasmid (67). Genes were ectopically expressed from the *pilA* promoter in plasmids integrated by site-specific recombination at the *attB* site. All plasmids were verified by sequencing. All strains were confirmed by PCR. Oligonucleotides are listed in *SI Appendix*, Table 3. *M. xanthus* suspension cultures were grown in 1% CTT broth (1% Bacto Casitone [Gibco], 10 mM Tris⋅HCl pH 8.0, 1 mM KPO_4_ pH 7.6, 8 mM MgSO_4_) or on 1% CTT 1.5% agar plates. When required, media were supplemented with kanamycin (50 µg mL^−1^) or oxytetracyclin (10 µg mL^−1^).

## Supplementary Material

Supplementary File

## Data Availability

All study data are included in the article and/or supporting information.
