# Supplementary material for: A noncanonical cytochrome c stimulates calcium binding by PilY1 for type IVa pili formation
Source: Proc Natl Acad Sci U S A. 2022 Feb 4;119(6):e2115061119. doi: 10.1073/pnas.2115061119 (PMC8833165; doi:10.1073/pnas.2115061119)
Supplement: Supplementary File [file pnas.2115061119.sapp.pdf]

PNAS
www.pnas.org

Supplementary Information

A non-canonical cytochrome *c* stimulates calcium binding by PilY1 for type IVa pili formation

Marco Herfurth, Anke Treuner-Lange, Timo Glatter, Nadine Wittmaack, Egbert Hoiczyk, Antonio J. Pierik and Lotte Søgaard-Andersen

This PDF file includes:

- Supplementary Materials & Methods
- Supplementary Figures 1-9
- Supplementary Tables 1-4
- Supplementary References

Corresponding author: Lotte Søgaard-Andersen[1]

E-mail: sogaard@mpi-marburg.mpg.de

Supplementary Materials & Methods

Bacterial strains and growth media. *E. coli* strains were grown in LB broth (1). Plasmids were propagated using *E. coli* NEB-Turbo. All media and buffers were prepared with ultrapure water using an ELGA Pureflex 2, that reduces the calcium concentration to <2.5 nM according to the manufacturer.

Bioinformatics. Homologs of TfcP were searched using BlastP (2). Pairwise sequence alignments were calculated using EMBOSS-Needle (3). Protein domains were identified using InterPro (4). Alignments were computed using MUSCLE (3). The homology model of PilY1.1 was generated using the Phyre2 server (5). Protein structure of TfcP was predicted using AlphaFold2 (6) and ColabFold (7). Ligand prediction was done using COACH (8). Structures were superimposed using PyMol (http://www.pymol.org/pymol).

Motility assay. T4aPdM was assayed as described (9). Briefly, exponentially growing *M. xanthus* cultures were harvested and concentrated in 1% CTT to a density of 7×10^9 cells ml^{-1}. 5 µl of the concentrated cell suspension were spotted on soft-agar CTT plates (0.5% CTT, 10 mM Tris-HCl pH 8.0, 1 mM KPO_4 pH 7.6, 8 mM $MgSO_4$, 0.5% select-agar (Invitrogen)) and incubated at 32°C for 24 hrs. Colonies were imaged using a Leica MZ75 stereomicroscope equipped with a Leica MC120 HD camera.

T4aP shearing assay. T4aP were sheared off *M. xanthus* cells as described (10). Briefly, cells were grown on CTT 1.5% agar plates at 32°C for three days, then scraped off, and resuspended in pili resuspension buffer (100 mM Tris-HCl pH 7.6, 150 mM NaCl) (1 ml per 60 mg cells). Cell suspensions were vortexed 10 min at maximum speed. A 100 µl aliquot was harvested and resuspended in 200 µl sodium dodecyl sulfate (SDS) lysis buffer (50 mM Tris-HCl pH 6.8, 2% SDS, 10% glycerol, 0.1 M DTT, 1.5 mM EDTA, 0.001% Bromophenol Blue), and denatured at 95°C for 10 min and used to determine the cellular PilA amount. The remaining cell suspension was cleared three times by 20 min centrifugation at 20,000 *g* at 4°C. Pili in the cleared supernatant were precipitated by adding 10× pili-precipitation buffer (final concentration: 100 mM $MgCl_2$, 2% PEG 6000, 100 mM Tris-HCl pH 7.6, 150 mM NaCl), incubation on ice for 4 hrs and centrifugation at 20,000 *g* for 30 min, 4°C. The pellet was resuspended in 1 µl SDS lysis buffer per mg cells and boiled for 10 min at 95°C. The samples were separated by SDS-PAGE and analysed for PilA accumulation by immuno-blot using PilA antibodies.

Immuno-blot and peroxidase staining. Immuno-blot analysis was carried out as described (1). Samples were prepared by harvesting exponentially growing *M. xanthus* cells and subsequently resuspension in SDS lysis buffer to an equal concentration of cells. Immuno-blot was done using as primary antibodies α-PilB, α-PilC, α-PilQ (11), α-PilA, α-LonD (12), α-

FLAG (Rockland; 600-401-383), α-GFP (Roche; 11814460001), α-MalE (New England Biolabs), α-PilY1.1 (13) and α-TfcP. Antibodies against TfcP were generated by Eurogentec against TfcP$^{\Delta 1\text{-}18}$-His$_6$ purified from *E. coli* Rosetta 2(DE3) containing plasmid pMH6 using native Ni-NTA affinity purification. As secondary antibodies, goat α-rabbit immunoglobulin G peroxidase conjugate (Sigma-Aldrich, A8275) and sheep α-mouse immunoglobulin G peroxidase conjugate (Amersham, NXA931) were used. Antibodies and conjugates were used in the following dilutions: 1:500 α-TfcP; 1:1000 α-PilY1.1; 1:3000 α-PilB; α-PilC; 1:5000 α-PilQ; α-PilA; 1:6000 α-LonD; 1:2000 α-GFP, α-MalE, α-FLAG and α-mouse peroxidase conjugate; and, 1:10,000 α-rabbit peroxidase conjugate. Blots were developed using Luminata™Western HRP substrate (Millipore). Unless otherwise noted, protein from 3×10^8 cells were loaded per lane. For peroxidase staining, protein was separated by SDS-PAGE, blotted on a nitrocellulose membrane and developed using Luminata™Western HRP substrate.

Fractionation of *M. xanthus*. *M. xanthus* was fractionated into fractions enriched for soluble, IM and OM proteins as described (14). Briefly, an exponentially growing *M. xanthus* culture was harvested and the pellet resuspended in lysis buffer (50 mM Tris-HCl pH7.6, Protease inhibitor cocktail (Roche)) (1 ml per 80 mg cells). A 75 µl aliquot was taken as the whole cell sample, suspended with SDS-lysis buffer and boiled 10 min at 95°C. Cells were lysed using sonication and lysates cleared by centrifugation at 8000 *g* for 1 min. The cleared lysate was subjected to ultra-centrifugation using an Air-Fuge (Beckman) at ~150,000 *g* for 1 hr. The resulting supernatant contains soluble proteins and was mixed with SDS-lysis buffer. The pellet was resuspended in detergent-lysis buffer (50 mM Tris-HCl pH 7.6, 2% Triton X-100) and subjected to ultra-centrifugation as described. The resulting supernatant is enriched for IM proteins while the pellet is enriched for OM proteins. The supernatant was mixed with SDS lysis buffer and the pellet resuspended in SDS-lysis buffer. The samples were analysed by SDS-PAGE and immuno-blot.

To identify proteins enriched in the periplasm, 100 ml of exponentially growing *M. xanthus* cells inCTT suspension culture were harvested and resuspended in 300 µl TSE8-buffer (200 mM Tris-HCl pH 8.0, 1 mM EDTA, 20% sucrose) supplemented with a Protease inhibitor cocktail (Roche) (15). Cells were incubated on a rotary shaker for 30 min at 4°C and harvested at 16,000 *g,* 4°C for 30 min. The supernatant containing periplasmic proteins was subjected to ultracentrifugation using an Air-Fuge at 150,000 *g* for 30 min. The cleared supernatant was precipitated with acetone and resuspended in SDS-lysis buffer.

Fluorescence microscopy. Exponentially growing *M. xanthus* cells were spotted on 1% agarose pads supplemented with TPM (10 mM Tris-HCl pH 8.0, 1 mM KPO_4 pH 7.6, 8 mM

$MgSO_4$) and incubated for 30 min at 32°C before microscopy. Cells were imaged using a Leica DMI600B microscope with a Hamamatsu Flash 4.0 camera. Images were recorded with Leica MM AF software and processed with Metamorph.

Targeted proteomics. To identify peptides of T4aPM proteins suitable for targeted-mass spectrometry (MS) analysis, we performed sample preparation on *M. xanthus* cell pellets for total proteome analysis as described (12). Briefly, proteins were extracted from cell pellets by heat exposure in the presence of 2% sodium-lauroylsarcosinate. Extracts were then reduced, alkylated and digested overnight using trypsin (Promega). Peptides were purified using C18 solid phase extraction and analysed on a Q-Exactive Plus mass spectrometer connected to an Ultimate 3000 RSLC and a nanospray flex ion source (all Thermo Scientific). The peptides were analysed using data dependent acquisition with settings as described (12). MS raw data were searched using Mascot (Matrix Science) and loaded into Scaffold 4 (Proteome software) for further data evaluation. Peptides considered most amenable for targeted MS were chosen for reference peptide synthesis (JPT Peptide Technologies, Berlin) containing heavy labelled (^{13}C and ^{15}N) C-terminal Lys or Arg residues with a resulting mass shift of +8 Da and +10 Da, respectively. Sequences of reference peptides are listed in Supplementary Table 4. For targeted MS experiments, reference peptides and iRT retention calibration peptides (Biognosys) were spiked into the *M. xanthus* total proteome peptide samples (generated as described), and analysed by liquid chromatography (LC)-MS.

Peptides were separated on a 90 min gradient from 2-50% acetonitrile at a flow rate of 300 nl min^{-1}, and analysed by MS in targeted parallel reaction monitoring (PRM) mode. The mass spectrometer first acquired a full MS-Selected Ion Monitoring (SIM) scan with an MS1 resolution of 70,000, AGC (automatic gain control) target setting of $1e^6$ and 100 ms max injection time. Then PRM scans were carried out with a MS2 resolution of 35,000, AGC target setting of $2e^5$, 100 ms maximum injection time with a quadrupole isolation window of 1.6 m/z. Normalised collision energy was set to 27%. All stages of targeted MS data analysis was carried out in Skyline (20.2.1.384) (16). Results with dot-product <0.85 or $ratio_{heavy/light}$<0.005 were excluded from the analysis.

Proteome analysis of T4aP. Label-free quantification (LFQ) MS of the pili proteome was carried out as described (12). Briefly, pili were purified following the shearing assay protocol with the modification that after precipitation, pili were resuspended in pili-resuspension buffer and re-precipitated with pili-precipitation buffer three times. Pili were resuspended in pili-resuspension buffer to 1 µl buffer per 1 mg cells. 25% of the pili sample was mixed with SDS-lysis buffer and analysed by SDS-PAGE and subsequent staining with Coomassie

Blue. The remaining 75% were precipitated with acetone. The dried acetone pellets were resuspended, reduced, alkylated and digested with trypsin as described (12). Pili LFQ proteomics analysis was carried out on an Exploris 480 mass spectrometer (Thermo Scientific), connected to an Ultimate 3000 RSLC. Peptides were separated on a 60 min gradient from 2-50% acetonitrile at a flow rate of 300 nl min^{-1}. The Exploris 480 mass spectrometer first acquired a full MS scan with an MS1 resolution of 60,000, AGC target setting of 3e^{6} and 60 ms max injection time, followed by MS/MS scans of Top-20 most abundant signals. For MS/MS scans a resolution of 7,500 was set, with an AGC of 2e^{5} and 30 ms max. injection time. Normalised collision energy was set to 27% and the isolation window of the quadrupole was 1.6 m/z. All MS raw data was analysed by MaxQuant (1.6.17.0). iBAQ values were calculated as described (12) as the sum of all peptide intensities for a given protein divided by the number of theoretically MS observable peptides. Following MaxQuant analysis, the iBAQ values were normalised by the total iBAQ sum independently of the highly abundant PilA.

Purification of MalE-TfcP. For purification of MalE-TfcP/MalE-TfcP^{C91M}, gene expression was done in *E. coli* strain BL21 containing the helper plasmid pEC86, which encodes the *ccm* genes for cytochrome *c* maturation of *E. coli*, as well as pMH31 (MalE-TfcP) or pMH39 (MalE-TfcP^{C91M}) using auto-induction in buffered 5052-Terrific-Broth (0.5% glycerol, 0.05% glucose, 0.2% lactose, 2.4% yeast extract, 2% tryptone, 25 mM Na_2HPO_4, 25 mM KH_2PO_4, 50 mM NH_4Cl, 5 mM Na_2SO_4, 2 mM $MgSO_4$) (17) containing chloramphenicol (25 µg ml^{-1}) and carbenicillin (100 µg ml^{-1}). After 24 hrs incubation at 37°C, cells were harvested, and resuspended in MBP-lysis buffer (100 mM Tris-HCl pH 7.0, 200 mM NaCl) supplemented with EDTA-free protease inhibitor cocktail (Roche) and lysed using sonication. The lysate was cleared by centrifugation at 20,000 *g*, 4°C for 30 minutes and loaded onto a 5 ml HighTrap MBP column (GE Healtcare) using an Äkta-Pure system (GE Healthcare). The column was washed with lysis buffer and protein eluted with 10 column volumes MBP-elution buffer (100 mM Tris-HCl pH 7.0, 200 mM NaCl, 10 mM maltose). The elution fractions containing MalE-TfcP/MalE-TfcP^{C91M} were pooled and diluted four fold in 100 mM Tris-HCl pH 7.0. The pooled and diluted samples were loaded onto a HighTrap SP ion exchange column. The column was washed with IEX-wash buffer (100 mM Tris-HCl pH 7.0) and protein eluted in a linear gradient with IEX-elution buffer (100 mM Tris-HCl pH 7.0, 2 M NaCl). Samples were concentrated using an Amicon Ultra filter with 10 kDa cutoff and loaded on a HiLoad 16/600 Superdex 200 pg (GE Healthcare) size exclusion chromatography column equilibrated with SEC-buffer (50 mM Tris-HCl pH 7.6, 50 mM NaCl). Protein was either used fresh or snap-frozen in buffer containing SEC-buffer with 10% glycerol.

Purification of TfcP. For purification of tag-free TfcP, *E. coli* BL21 was transformed with plasmids pMH41 and pEC86 and synthesis of TfcP induced using auto-induction in buffered 5052-Terrific-Broth as described for MalE-TfcP. pMH41 encodes untagged TfcP in which the native type I signal peptide is replaced with the MalE signal peptide for efficient secretion to the periplasm. Expression cultures were grown at 37°C until reaching turbidity and further incubated at 22°C over-night. Cells were harvested and the periplasmic fraction extracted using the TSE-method as described (15). In brief, cells were resuspended in TSE7-buffer (200 mM Tris-HCl pH 7, 1 mM EDTA, 20% Sucrose) and incubated 30 min following by harvesting of cells 30 min at 16,000 *g*. Subsequently, periplasmic proteins are present in the supernatant. TfcP was enriched from the periplasmic fraction using a HiTrap SP HP column on an Äkta pure system using the same buffers as for MalE-TfcP. Fractions were pooled, concentrated and loaded on a Superdex75 10/300 size exclusion chromatography column (GE Healthcare) equilibrated with SEC-buffer (50 mM Tris-HCl pH 7.6, 50 mM NaCl).

UV-Vis spectroscopy. UV-Vis measurements of purified (oxidised) and reduced MalE-TfcP/MalE-$TfcP^{C91M}$ was conducted on a Tecan M200Pro platereader or a Shimadzu 1900 spectrophotometer. Protein was diluted to an absorbance of ~0.7. After measurement of the oxidised spectrum, protein was reduced by adding a few crystals of sodium-dithionite, equilibrated for 15 min and the reduced spectrum recorded.

Redox titrations. Redox titrations were carried out in a Coy anaerobic tent (3% H_2, <5 ppm O_2). MalE-TfcP in HEPES buffer, pH 7.0, was mixed with 20 µM (final concentration) of the following redox mediators: Phenosafranin, safranin T, neutral red, benzyl viologen, and methyl viologen. The solution potential was measured with an InLab redox micro combination electrode (Mettler Toledo) under anaerobic conditions. Correction to redox potentials vs. H_2/H^+ was done by addition of 207 mV to the reading of the potentiometer. Stirring was done using a 8 mm teflon coated stirrer bar. For redox titration using visible spectroscopy (using a Shimadzu 1900 spectrophotometer), automated addition of 15 µl buffered 0.2 mM sodium-dithionite solution was done using a remotely controlled peristaltic pump (Pharmacia P1) for 60 sec followed by 2 min equilibration and 2 min recording of the spectra in the 600-460 nm range. The normalised absorbance increase at 550 nm (corrected by the absorbance for titration of mediators only) was fitted to the Nernst equation for n=1 at 298 K. For the EPR titration, manual addition of aliquots of buffered sodium-dithionite was used. After stabilisation of the solution potential, 300 µl samples were withdrawn, transferred to EPR tubes, which were capped with rubber tubing and an acrylic glass stick. Samples were shock-frozen and stored in liquid nitrogen until the EPR measurements.

EPR spectroscopy. EPR spectra were recorded with an X-band EPR spectrometer (Bruker Elexsys E580) in a 4122HQE-W1/1017 resonator. The temperature of the samples in Ilmasil PN quartz tubes (4.7±0.2 mm outer diameter, 0.45±0.05 mm wall thickness) was maintained at 12 K with an ESR900 continuous flow helium cryostat (Oxford Instruments). The modulation frequency was 100 kHz and the modulation amplitude 1.5 mT. Spectra were averages for four 90 sec scans. For the titration, the normalised amplitude of the derivative-shaped feature of the low spin EPR signal of the ferric state at g=2.26 was used for a fit to the Nernst equation (n=1, T=298 K).

Operon mapping. Total RNA was isolated from exponentially growing *M. xanthus* cultures using the Monarch Total RNA Miniprep Kit (NEB). 10^9 cells were harvested and resuspended in 200 µl lysis-buffer (100 mM Tris-HCl pH 7.6, 1 mg ml^{-1} lysozyme). After incubation at 25°C for 5 min cells were lysed and RNA purified according to manufacturer's protocol with the exception that the on-column DNase treatment was omitted. RNA was eluted in RNase-free water and subsequently treated with Turbo DNase and purified using the Monarch RNA Cleanup Kit (50 µg) (NEB) and eluted in RNase-free water. 1 µg of RNA was used for cDNA synthesis using the LunaScript RT SuperMix Kit (NEB) with and without reverse transcriptase (RT). cDNA was diluted 1:5 with water and 1 µl of diluted cDNA used for PCR reactions.

qRT-PCR. For qRT-PCR RNA was isolated and cDNA synthesised as described for operon mapping. qPCRs were carried out using the Luna Universal qPCR MasterMix (NEB) with the primers listed in Supplementary Table 3 and measured on an Applied Biosystems 7500 Real-Time PCR system. Relative gene expression levels were calculated using the comparative C_T method (18). *Mxan_3298* (*tuf2*), which encodes elongation factor Tu, and *mxan_3303* (*rpsS*), which encodes the small ribosomal subunit protein S19, were used as internal controls. All experiments were done with three biological replicates and two technical replicates.

Growth curve. Three independent precultures were diluted to an OD_{550} 0.05 and incubated at 32°C shaking for 72 hrs. Growth was monitored by taking samples at indicated time points and measuring the OD_{550}.

Calcium binding assay. Calcium binding of TfcP was assayed as described (19). Briefly, purified TfcP and bovine heart cytochrome *c* (Sigma) was passed over a Chelex-100 column (Bio-Rad) and subsequently buffer exchanged using a PD MiniTrap G-25 equilibrated with 50 mM Tris pH7.6; 50 mM NaCl. Protein was serially diluted and titration reactions with 20 µM OregonGreen-BAPTA5N, 2 µM $CaCl_2$ measured on a Tecan M200. Binding curves were fitted using GraphPad Prism.

Statistics and reproducibility. Data shown for operon mapping, T4aP-dependent motility, T4aP shearing assays, immuno-blot experiments, UV-Vis spectroscopy, growth curves, size exclusion chromatography, and fluorescence microscopy were obtained in at least two biological replicates with similar results. For targeted proteomics and LFQ-analysis of the pili proteome, four biological replicates were analysed. qRT-PCR analysis were conducted with three biological replicates each with two technical replicates. Calcium-binding, redox titrations and EPR-spectroscopy where done in a single experiment.

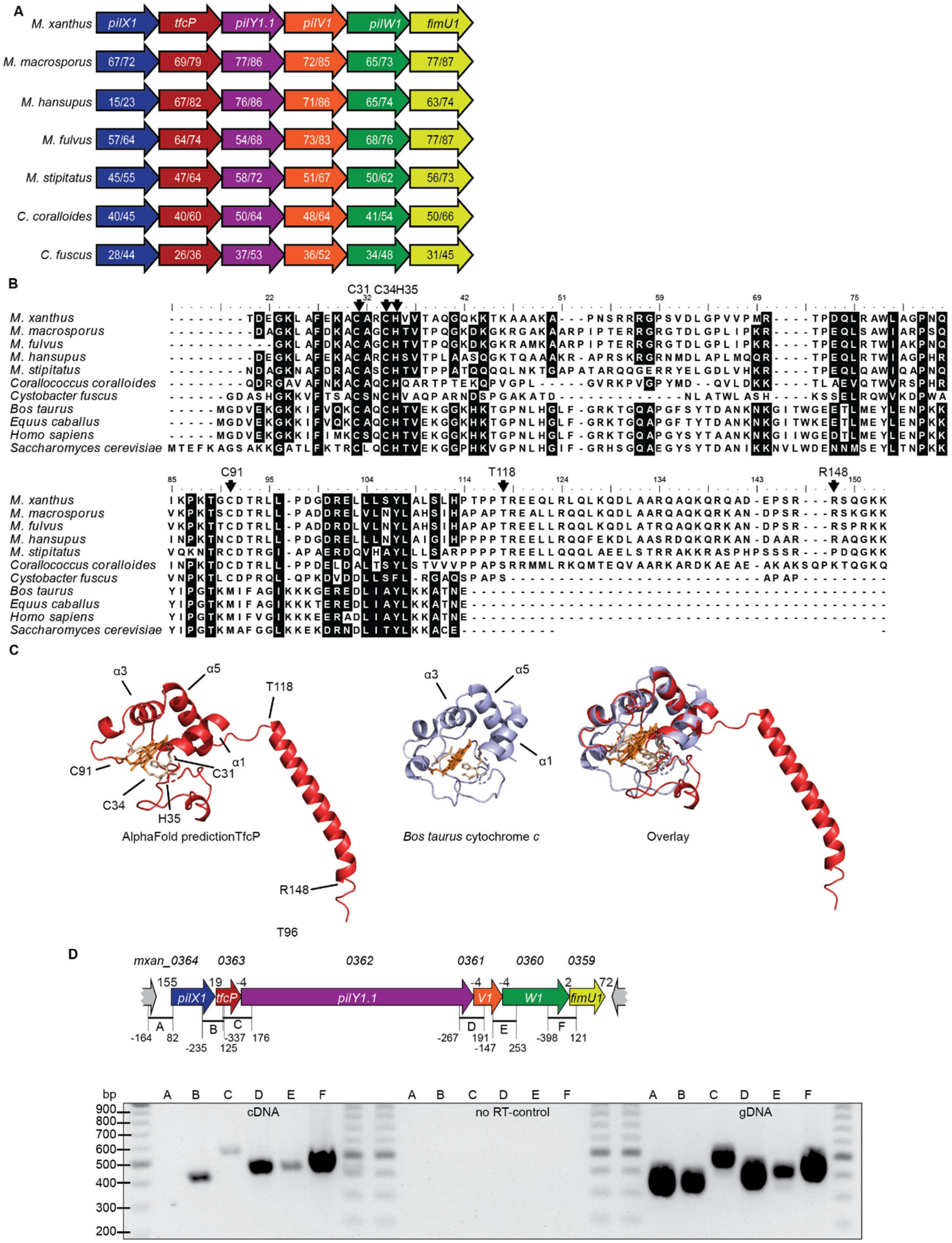

Figure S1. Analysis of cluster_1 and TfcP.

A Comparison of cluster_1 gene clusters in myxobacteria. All six proteins in the listed species are encoded at the same locus. Arrows indicate direction of transcription. Numbers within genes represent identity/similarity determined by pairwise alignment with the respective *M. xanthus* protein. **B** Multiple sequence alignment of TfcP and homologs as well as canonical cytochromes *c*. Shading represents >70% conservation. The cytochrome *c*

signature motif CxxCH and the distal Cys^{91} residue in TfcP are indicated. T118 and R148 indicate the N-terminal and C-terminal residues in the C-terminal α-helix. Numbering of amino acids is according to the unprocessed, full-length TfcP protein. **C** Structure of TfcP predicted by AlphaFold2 with the cytochrome *c* signature motif CxxCH and the distal Cys^{91} residue indicated (left), crystal structure of *Bos taurus* cytochrome *c* (PDB 2B4Z) (middle) and overlay of the TfcP AlphaFold2 model (red) with the crystal structure of *B. taurus* cytochrome *c (*blue*)* (right). Orange: Heme; brown: Heme-binding residues; α1, α3 and α5 make up the typical cytochrome *c* fold (20). **D** Operon mapping of cluster_1 in *M. xanthus*. Upper panel, genetic organization of cluster_1. Locus tags are included above genes and gene names within genes. Distances between start and stop codons are shown above. Letters below arrows indicate the fragments amplified by PCR. Numbers indicate the distance from the 5'-end of a primer to the first base of the stop codon or the first base of the start codon as appropriate. The PCR products amplified using cDNA, an enzyme free reverse transcription reaction and genomic DNA as templates were separated on a 1% agarose gel. Letters above the individual lanes correspond to the letters of the primer combinations depicted above. Molecular size markers in base-pairs are shown on the left.

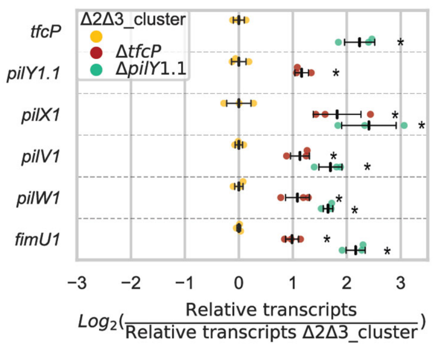

Figure S2. qRT-PCR analysis of transcript levels of cluster_1 genes.
Total RNA was isolated from cells grown in 1.0% CTT suspension culture. Individual data points represent three biological replicates with each two technical replicates, and in which the ratio of the relative transcript level in a mutant over the transcript level in the $WT_{\Delta 2\Delta 3}$ strain is plotted. Center marker and error bars: Mean and STDEV. Statistical analyses were performed as in Fig. 3A. *,P< 0.05.

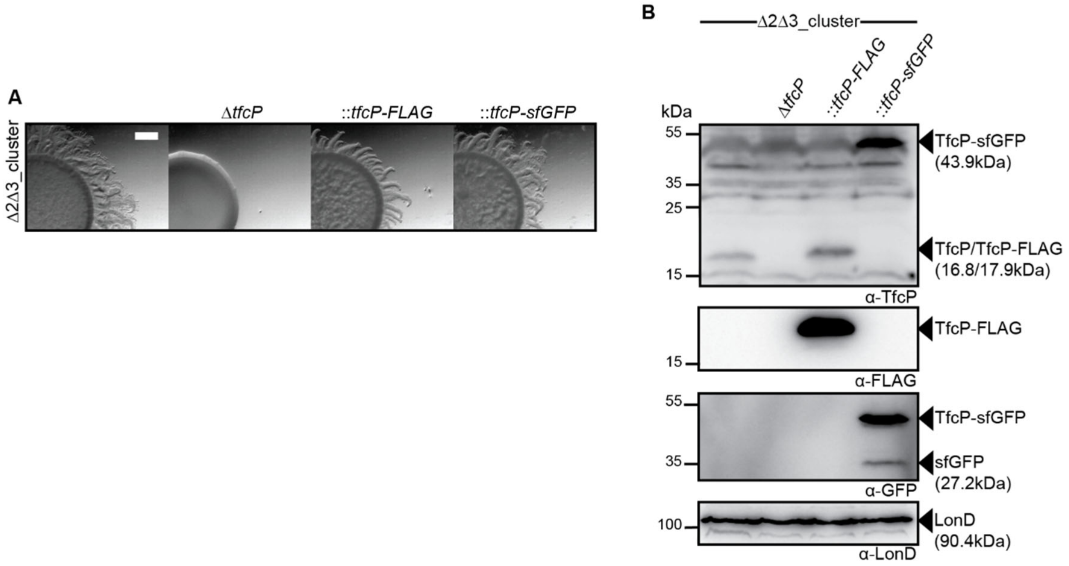

Figure S3. Analysis of TfcP-FLAG and TfcP-GFP.
A Assay for T4aPdM. Strains were assayed as in Fig. 2A. Scale bar, 1 mm. **B** Immuno-blot analysis of TfcP-FLAG and TfcP-sfGFP accumulation. Cells were grown in 1.0% CTT suspension culture and analysed as in Fig. 3B. Note that immuno-blots probed with α-TfcP contain a cross-reacting band at the size of TfcP-sfGFP.

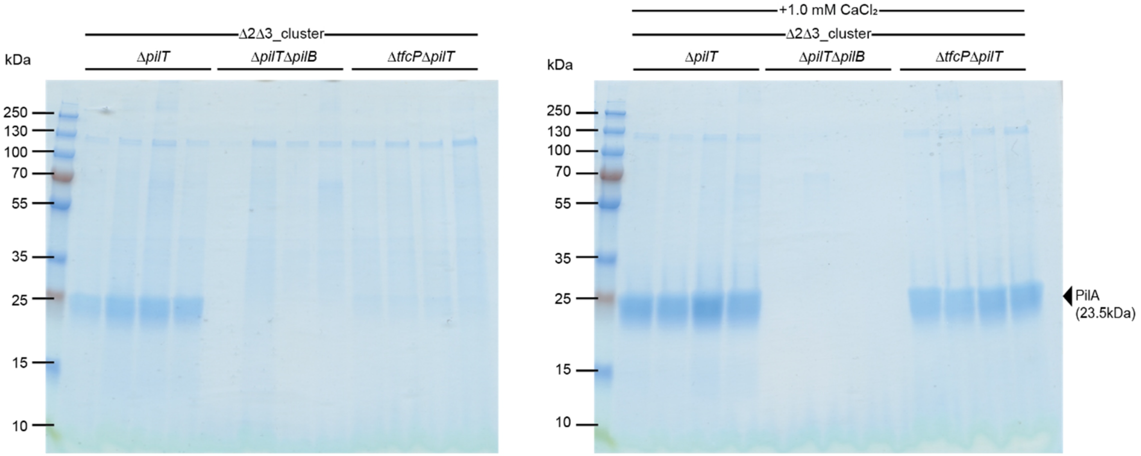

Figure S4. Analysis of purified pili
Pili from 15 mg of cells of the indicated strains were loaded on SDS-PAGE and stained with Coomassie Blue. The left and right gels show pili from cells grown on 1.5% agar supplemented with 1.0%CTT in the absence and presence of 1.0 mM additional $CaCl_2$, respectively.

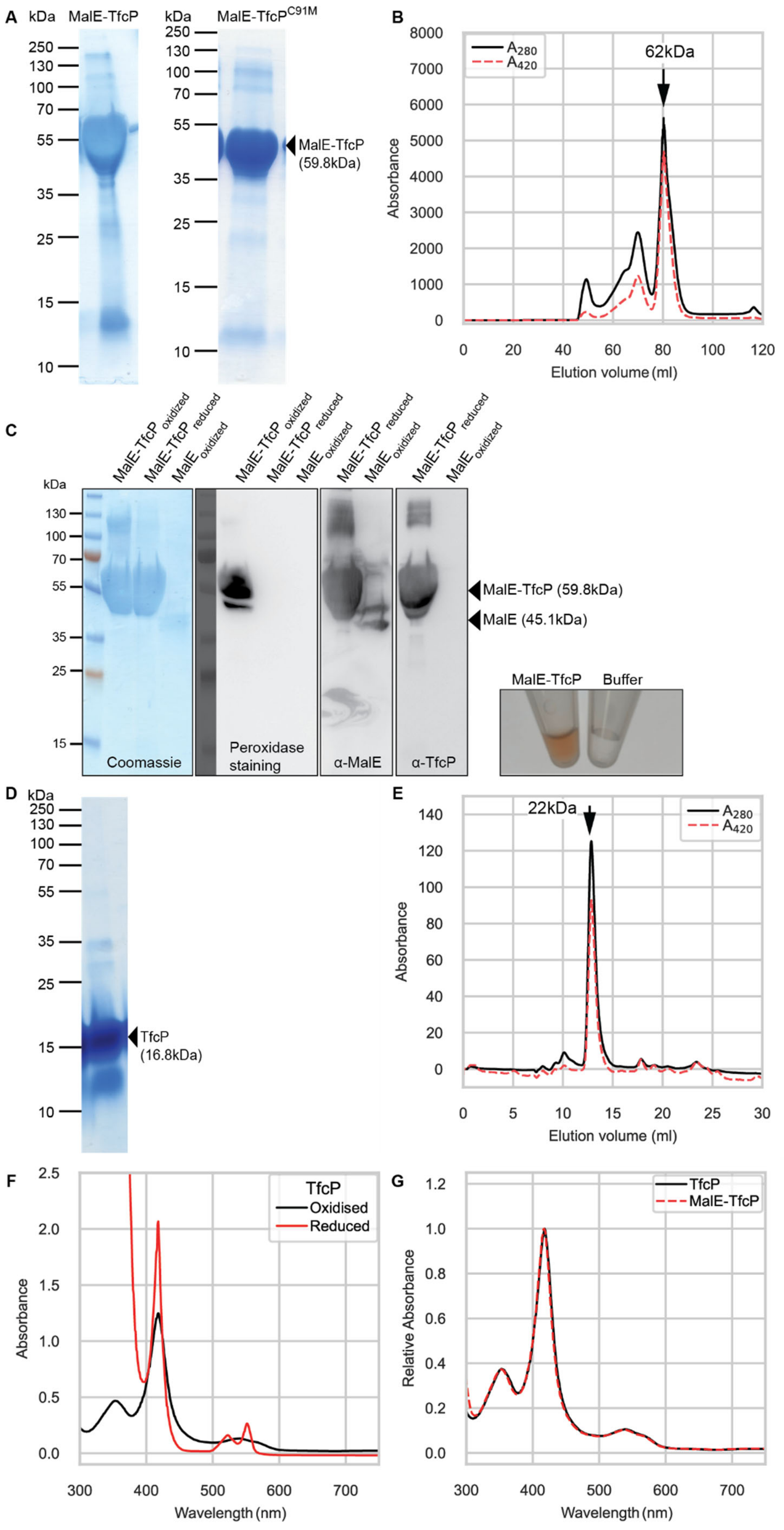
A
kDa
MalE-TfcP
MalE-TfcP C91M
MalE-TfcP (59.8kDa)
B
62kDa
Absorbance
Elution volume (ml)
C
MalE-TfcP oxidized
MalE-TfcP reduced
MalE oxidized
Coomassie
Peroxidase staining
α-MalE
α-TfcP
MalE-TfcP (59.8kDa)
MalE (45.1kDa)
MalE-TfcP
Buffer
D
TfcP (16.8kDa)
E
22kDa
Absorbance
Elution volume (ml)
F
TfcP
Oxidised
Reduced
Absorbance
Wavelength (nm)
G
TfcP
MalE-TfcP
Relative Absorbance
Wavelength (nm)

Figure S5. *In vitro* analysis of TfcP

A Purified MalE-TfcP variants used for spectroscopic analysis. ~5 µg of purified proteins were separated by SDS-PAGE and stained by Coomassie Blue. The fusions for overexpression contains the MalE type I signal peptide and lacks the TfcP signal peptide. The molecular mass is indicated for full-length unprocessed proteins. **B** Absorbance at 280 nm and 420 nm during size exclusion chromatography of MalE-TfcP. Calculated molecular weight of MalE-TfcP based on the elution volume is indicated. **C** TfcP heme-binding assay. Panels from left-to-right**,** MalE-TfcP in oxidised (as purified) and reduced state (after addition of DTT) stained with Coomassie Blue, analysed for heme-binding by peroxidase staining using a luminescent horse radish peroxidase (HRP) substrate and MalE as negative control, detected by immuno-blotting with α-MalE and α-TfcP as indicated, and image of purified MalE-TfcP in buffer. **D** Purified TfcP used for spectroscopic analysis and the calcium binding experiment. Molecular mass is indicated for the full-length unprocessed protein. **E** Absorbance at 280 nm and 420 nm during size exclusion chromatography of TfcP. Calculated molecular weight of TfcP based on the elution volume is indicated. **F** UV-Vis spectra of purified TfcP in the oxidised and reduced state. Experiments were performed on a Tecan200Pro platereader. **G** Comparison of UV-Vis spectra of MalE-TfcP and TfcP. Spectra were normalized to the Soret-Peak.

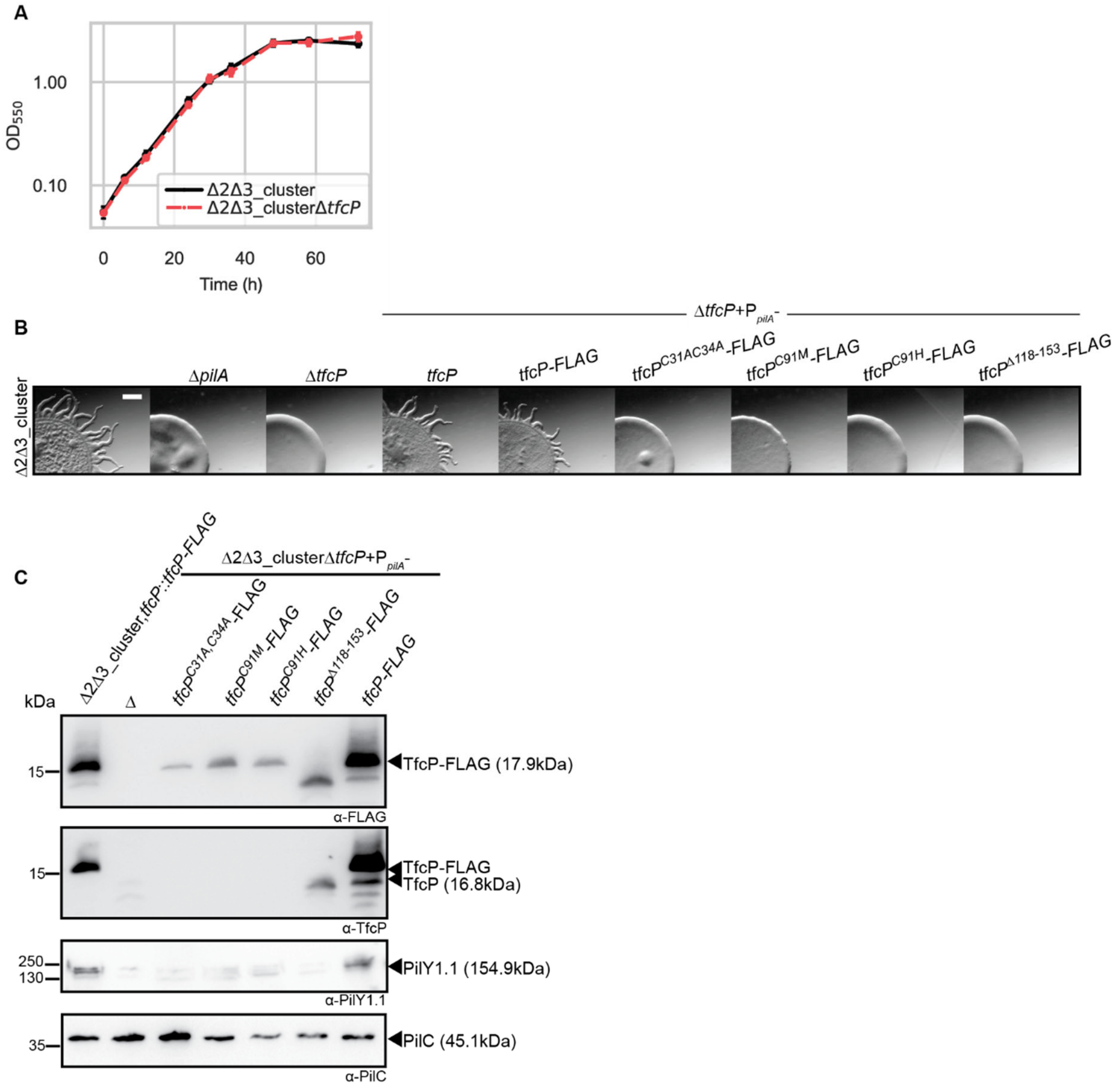

Figure S6. Amino acid substitution in heme-binding residues of TfcP or deletion of the C-terminal extension affect protein stability

A Growth curves of $WT_{\Delta2\Delta3}$ and $WT_{\Delta2\Delta3}\Delta$*tfcP*. Points represent mean and STDEV from three biological replicates. **B** Assays for T4aPdM. $WT_{\Delta2\Delta3}$ and strains expressing mutant TfcP variants were spotted on 0.5% agar supplemented with 0.5% CTT and imaged after 24 hrs. Scale bar, 1 mm. **C** Accumulation of TfcP and PilY1.1 in strains expressing TfcP variants. Protein from the same number of cells grown in 1.0% CTT suspension culture was separated by SDS-PAGE and analysed by immuno-blotting. The lane labeled with Δ contains whole cell lysate of a Δ1Δ2_cluster strain as a negative control. PilC was used as a loading control.

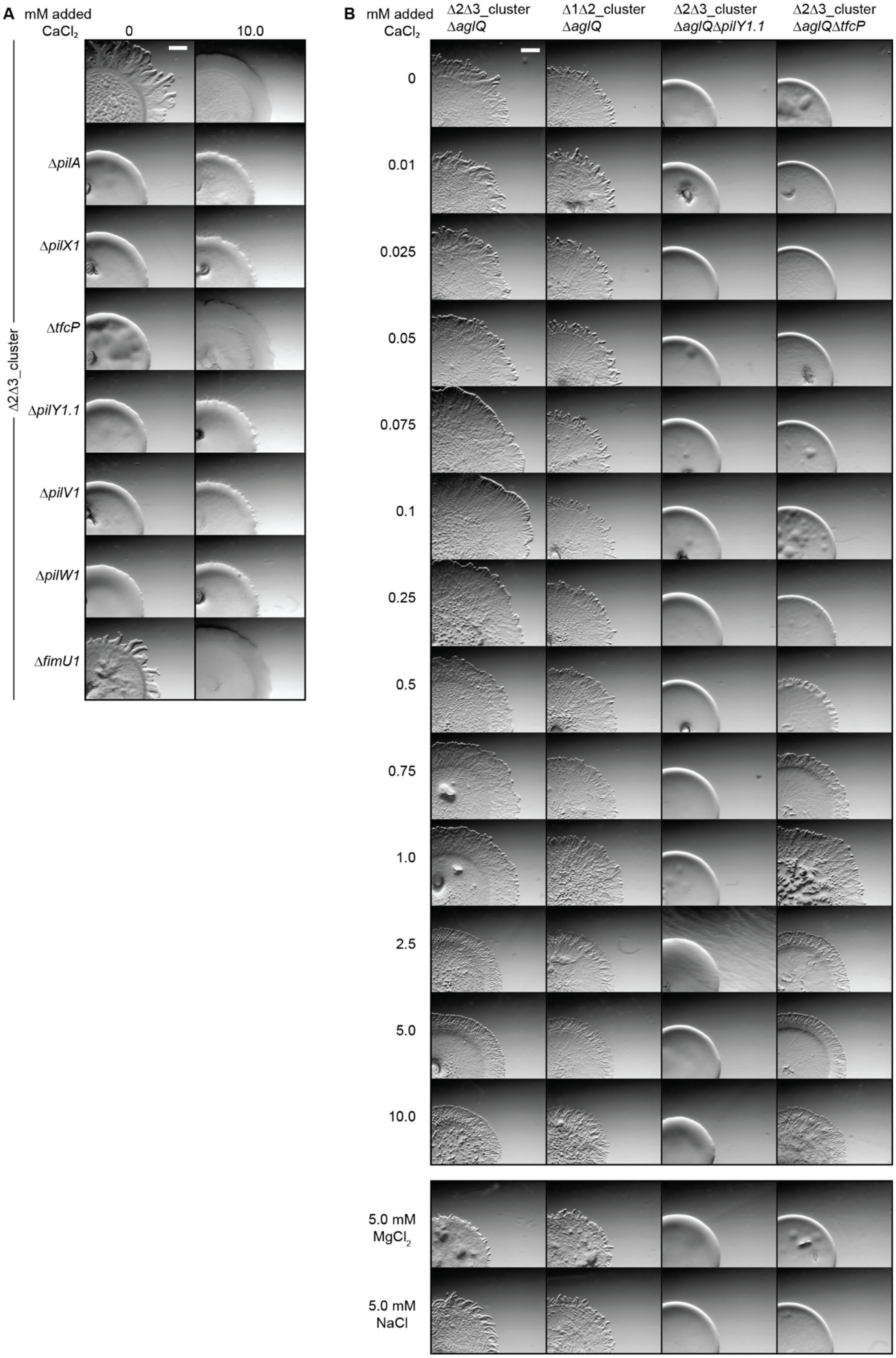

Figure S7. $CaCl_2$ affects T4aPdM of *M. xanthus*

A Assay for T4aPdM. Cells were grown in 1.0% CTT suspension culture and plated on 0.5% agar supplemented with 0.5% CTT and imaged after 24 hrs. The final concentration of added $CaCl_2$ is indicated. Scale bar, 1 mm. **B** Assay for T4aPdM. Cells were grown in 1.0% CTT suspension culture and plated on 0.5% agar supplemented with 0.5% CTT and imaged after 24 hrs. The final concentration of added $CaCl_2$, $MgCl_2$ and NaCl is indicated. Scale bar, 1 mm.

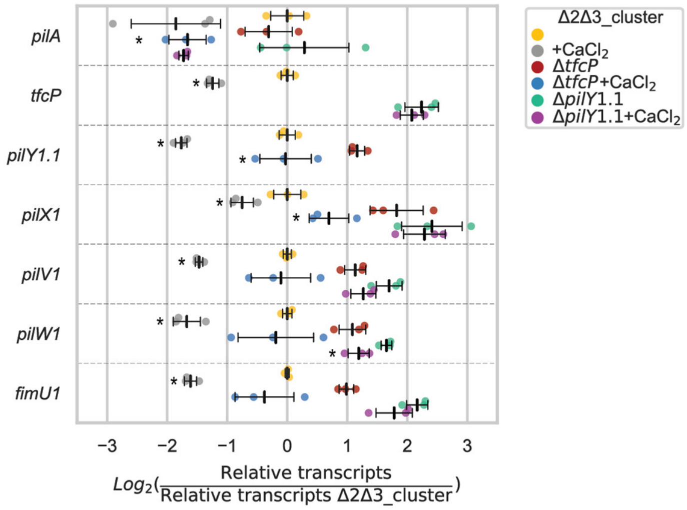

Figure S8. qRT-PCR analysis of transcript levels of cluster_1 genes and *pilA*.
Total RNA was isolated from cells grown in 1.0% CTT suspension culture without or with added calcium as indicated. Individual data points represent three biological replicates with each two technical replicates, and in which the ratio of the relative transcript level in a mutant over the transcript level in the $WT_{\Delta 2\Delta 3}$ strain is plotted. Data for samples without added $CaCl_2$ are the same as in Fig. S2 and included for comparison. Center marker and error bars: Mean and STDEV. Statistical analyses were performed as in Fig. 6E. *,P< 0.05.

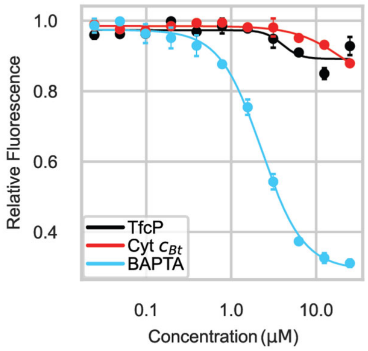

Figure S9. TfcP does not detectably bind calcium.
Calcium binding by purified TfcP was assayed by titration against a fluorescent calcium reporter. *Bos taurus* cytochrome *c* (Cyt C_{Bt}) was used as negative and BAPTA as positive control. Points represent mean and STDEV from two technical replicates.

Supplementary Table 1. Strains used in this study

Name	Description	Reference
	M. xanthus **strains**	
SA6892	Δ*cluster_2 (mxan_1021-1017)* Δ*cluster_3 (mxan_1369-1365)*	(12)
SA6888	Δ*cluster_1* (*mxan_0364-0359)* Δ*cluster_2*	(12)
SA8754	Δ*cluster_2* Δ*cluster_3* Δ*pilT* (*mxan_5787*) Δ*pilB* (*mxan_5788*)	This study
SA7717	Δ*cluster_2* Δ*cluster_3* Δ*pilT*	This study
SA7698	Δ*cluster_2* Δ*cluster_3* Δ*aglQ* (*mxan_6861*)	This study
SA7703	Δ*cluster_1* Δ*cluster_2* Δ*aglQ*	This study
SA7649	Δ*cluster_2* Δ*cluster_3* Δ*pilX1* (*mxan_0364*)	This study
SA7648	Δ*cluster_2* Δ*cluster_3* Δ*tfcP* (*mxan_0363*)	This study
SA7647	Δ*cluster_2* Δ*cluster_3* Δ*pilY1.1* (*mxan_0362*)	This study
SA7646	Δ*cluster_2* Δ*cluster_3* Δ*pilV1* (*mxan_0361*)	This study
SA7645	Δ*cluster_2* Δ*cluster_3* Δ*pilW1* (*mxan_0360*)	This study
SA7644	Δ*cluster_2* Δ*cluster_3* Δ*fimU1* (*mxan_0359*)	This study
SA7672	Δ*cluster_2* Δ*cluster_3* Δ*pilT* Δ*pilX1*	This study
SA7673	Δ*cluster_2* Δ*cluster_3* Δ*pilT* Δ*tfcP*	This study
SA7674	Δ*cluster_2* Δ*cluster_3* Δ*pilT* Δ*pilY1.1*	This study
SA7675	Δ*cluster_2* Δ*cluster_3* Δ*pilT* Δ*pilV1*	This study
SA7676	Δ*cluster_2* Δ*cluster_3* Δ*pilT* Δ*pilW1*	This study
SA7677	Δ*cluster_2* Δ*cluster_3* Δ*pilT* Δ*fimU1*	This study
SA9004	Δ*cluster_2* Δ*cluster_3 tfcP::tfcP-FLAG*	This study
SA9009	Δ*cluster_2* Δ*cluster_3 tfcP::tfcP-sfGFP*	This study
SA9012	Δ*cluster_2* Δ*cluster_3* Δ*pilY1.1* Δ*aglQ*	This study
SA9016	Δ*cluster_2* Δ*cluster_3* Δ*tfcP* Δ*aglQ*	This study
SA9017	Δ*cluster_2* Δ*cluster_3* Δ*pilA* (*mxan_5783*)	This study
SA9019	Δ*cluster_2* Δ*cluster_3* Δ*tfcP pilM (mxan_5776)::mCherry-pilM*	This study
SA7680	Δ*cluster_2* Δ*cluster_3* Δ*tfcP attB::*P_{pilA}*-tfcP*	This study
SA9040	Δ*cluster_2* Δ*cluster_3* Δ*tfcP attB::*P_{pilA} *-tfcP-FLAG*	This study
SA9041	Δ*cluster_2* Δ*cluster_3* Δ*tfcP attB::*P_{pilA} *-tfcP*^{C91H}*-FLAG*	This study
SA9042	Δ*cluster_2* Δ*cluster_3* Δ*tfcP attB::*P_{pilA} *-tfcP*^{C91M}*-FLAG*	This study
SA9043	Δ*cluster_2* Δ*cluster_3* Δ*tfcP attB::*P_{pilA} *-tfcP*$^{\Delta 118\text{-}153}$*-FLAG*	This study
SA9031	Δ*cluster_2* Δ*cluster_3* Δ*pilY1.1 attB::*P_{pilA} *-pilY1.1*	This study
SA9032	Δ*cluster_2* Δ*cluster_3* Δ*fimU1 attB::*P_{pilA} *-fimU1*	This study
SA9033	Δ*cluster_2* Δ*cluster_3* Δ*pilV1 attB::*P_{pilA} *-pilV1*	This study
SA9044	Δ*cluster_2* Δ*cluster_3* Δ*tfcP attB::*P_{pilA} *-tfcP*^{C31A,C34A}*-FLAG*	This study
SA9034	Δ*cluster_2* Δ*cluster_3* Δ*pilW1 attB::*P_{pilA} *-pilW1*	This study
SA9055	Δ*cluster_2* Δ*cluster_3* Δ*pilX1 attB::*P_{pilA} *-pilX1*	This study
SA9051	Δ*cluster_2* Δ*cluster_3 pilM::mCherry-pilM*	This study
SA9064	Δ*cluster_2* Δ*cluster_3 pilY1.1::pilY1.1*^{D1173A}	This study
SA9066	Δ*cluster_2* Δ*cluster_3* Δ*tfcP pilY1.1::pilY1.1*^{D1173A}	This study
SA9068	Δ*cluster_2* Δ*cluster_3* Δ*aglQ pilY1.1::pilY1.1*^{D1173A}	This study
SA9069	Δ*cluster_2* Δ*cluster_3* Δ*tfcP* Δ*aglQ pilY1.1::pilY1.1*^{D1173A}	This study
SA6024	Δ*pilBTCMNOPQ*	(21)
SA8721	Δ*pilQ; pilM::mcherry-pilM*	(12)

	E. coli **strains**	
NEB-Turbo	F' *proA*$^{+}$*B*$^{+}$ *lacI*^{q} Δ*lacZM15 / fhuA2* Δ*(lac-proAB) glnV galK16 galE15 R(zgb-210::Tn10)*Tet^{S} *endA1 thi-1* Δ*(hsdS-mcrB)5*	New England Biolabs
BL21	*fhuA2 [lon] ompT gal [dcm] ΔhsdS*	New England Biolabs

Supplementary Table 2. Plasmids used in this study

Name	Description	Reference
pBJ114	*galK* containing vector for generation of in-frame deletions in *M. xanthus*, Kan^R	(22)
pSW105	P_{pilA} , Kan^R, *attP*	(23)
pMal-p5x	Expression vector for periplasmic MalE fusions	NEB
pET24b+	Expression vector for His_6-tagged protein	Novagen
pMAT150	pBJ114, in-frame deletion of *pilT*	(12)
pBJd*aglQ*	pBJ114, in-frame deletion of *aglQ*	(24)
pMH12	pBJ114, endogenous *tfcP*-sfGFP	This study
pMH41	pMAL-p5x derived, MalE-signal peptide TfcP	This study
pMAT167	pBJ114, in-frame deletion of *pilX1*	(12)
pMAT164	pBJ114, in-frame deletion of *pilY1.1*	(12)
pMAT163	pBJ114, in-frame deletion of *pilB*	(12)
pMAT162	pBJ114, in-frame deletion of *pilA*	(12)
pMAT170	pBJ114, in-frame deletion of *pilB* and *pilT*	This study
pMAT336	pBJ114, endogenous mCherry-PilM	(12)
pMAT407	pSW105, P_{pilA} -*tfcP*-FLAG	This study
pMAT409	pSW105, P_{pilA} -$tfcP^{C91H}$-FLAG	This study
pMAT408	pSW105, P_{pilA} -$tfcP^{C91M}$-FLAG	This study
pMAT410	pSW105, P_{pilA} -$tfcP^{\Delta 118\text{-}153}$-FLAG	This study
pMAT210	pSW105, P_{pilA} -*pilY1.1*	This study
pMAT220	pSW105, P_{pilA} -*fimU1*	This study
pMAT222	pSW105, P_{pilA} -*pilV1*	This study
pMAT310	pSW105, P_{pilA} -*pilX1*	This study
pMH45	pSW105, P_{pilA} -$tfcP^{C31A,C34A}$-FLAG	This study
pMAT221	pSW105, P_{pilA} -*pilW1*	This study
pMH60	pBJ114, endogenous $pilY1.1^{D1173A}$	This study
pMH1	pBJ114, in-frame deletion of *tfcP*	This study
pMH2	pBJ114, in-frame deletion of *fimU1*	This study
pMH3	pBJ114, in-frame deletion of *pilW1*	This study
pMH4	pBJ114, in-frame deletion of *pilV1*	This study
pMH5	pET24b+, $tfcP\text{-}His_6$	This study
pMH7	pSW105, P_{pilA} -*tfcP*	This study
pMH10	pBJ114, endogenous *tfcP*-FLAG	This study
pMH31	pMAL-p5x, MalE-TfcP	This study
pMH39	pMAL-p5x, $MalE\text{-}TfcP^{C91M}$	This study
pMH45	pSW105, $tfcP^{C31A,C34A}$-FLAG	This study
pEC86	Constitutive expression of *ccm* genes of *E. coli*	(25)

Supplementary Table 3. Oligonucleotides used in this study

	Oligonucleotides used for cloning	
**Name**	**Sequence[1]**	**Brief description**
0359-A-HindIII	**GCGCAAGCTT**GCATGGTGACGCTGAGTCCC	Δ*fimU1*
0359-B-XbaI	**GCGCTCTAGAT**CCGCGTGTGTGCCTCATG	Δ*fimU1*
0359-C-XbaI	**GCGCTCTAGAT**GAGCACTGCCGGCACCTGAAG	Δ*fimU1*
0359-D-BamHI	**GCGCGGATCC**CGGAGGTGGAGCTGCTGC	Δ*fimU1*
0360-A-HindIII	**GCGCAAGCTT**AAGGTCTACGCGACCACGGC	Δ*pilW1*
0360-B-XbaI	**GCGCTCTAGA**CGTCTTCACGGCGCCATCCT	Δ*pilW1*
0360-C-XbaI	**GCGCTCTAGA**ACGGAAAATTGAGCATGAGG	Δ*pilW1*
0360-D-BamHI	**GCGCGGATCC**GGAAGTGGCGCAGGCCTTCG	Δ*pilW1*
0361-A-HindIII	**GCGCAAGCTT**CACGGGCTCTGGCATCGCCG	Δ*pilV1*
0361-B-XbaI	**GCGCTCTAGA**CGCTGTCACTGCGGCATCCT	Δ*pilV1*
0361-C-XbaI	**GCGCTCTAGA**ATGGCGCCGTGAAGACGACT	Δ*pilV1*
0361-D-BamHI	**GCGCGGATCC**CAGGTACTCCAGCGTCGGTA	Δ*pilV1*
0363-A-HindIII	**GCGCAAGCTT**GTGCCGCCGCTCAGGCATG	Δ*tfcP*
0363-Bflag-KpnI	**GCGCGGTACC**CTTCTTCCCCTGCGAACG	*tfcP-FLAG*
0363-Cflag-KpnI	**GCGCGGTACC**CTTCTTCCCCTGCGAACG	*tfcP-FLAG*
0363-B-XbaI	**GCGCTCTAGA**GATGAGTCGGTTCATGGG	Δ*tfcP*
0363-C-XbaI	**GCGCTCTAGA**TCGCAGGGGAAGAAGTGA	Δ*tfcP*
0363-D-BamHI	**GCGCGGATCC**GCGACAGGTTTCCGTAGG	Δ*tfcP*
sfGFP-B overlay	GCGCGGATGAGGGTGCGCATCATTTGTAGAGCTC	*tfcP-sfGFP*
0363-C overlay	TGATGCGCACCCTCATCCAGACACTGGCCG	*tfcP-sfGFP*
0363 AvaI FactorXa - SP	**GCGCCTCGGG**ATCGAGGGAAGGACGGATGAAGGCAAGCTCGCCTTC	MalE-TfcP/MalE-TfcP^{C91M}
0363 Stop HindIII	**CGCGAAGCTT**TCACTTCTTCCCCTGCGAACG	MalE-TfcP/MalE-TfcP^{C91M}/TfcP
MalE start NdeI*	**GCGCCATATG**AAAATAAAAACAGGTGCACGC	MalE-TfcP/MalE-TfcP^{C91M}
0363 Start XbaI	**GCGCTCTAGA**AACCGACTCATCCTGTTG	PpilA-tfcP
0363 nostop BamHI	**GCGCGGATCC**CTTCTTCCCCTGCGAACG	P_{pilA}-*tfcP*-FLAG/sfGFP
0363 Δ118-153 BamHI	**GCGCGGATCC**AGGAGGTGTGGGGTGGAGGCT	P_{pilA}-*tfcP*$^{\Delta 118\text{-}153}$-FLAG
0363-Bmut-XmaI	**GCGCCCCGGG**CGGCGGCCTTCTCGAAGGCGAG	*tfcP*^{C31A,C34A}
0363-Cmut-XmaI	**GCGCCCCGGG**CTCACGTCGTCACCGCGCAAG	*tfcP*^{C31A,C34A}
0363-Short-B	**GCGCTCTAGAG**TGGAGGCTGAGCGCCAG	*tfcP*$^{\Delta 118\text{-}153}$

0363-Short-C	**GCGCTCTAGA**TGATGCGCACCCTCATCCAGAC	*tfcP*$^{\Delta 118\text{-}153}$
PilY1_1 mut fwd HindIII	**GCGCAAGCTT**CAATCAAAACCAGATCAACAG	*pilY1.1*^{D1173A}
PilY1_1_mut rev BamHI	**GCGCGGATCC**GATGAACAAGTGATTGTCATG	*pilY1.1*^{D1173A}
0363-E	GTCTCTTGAGACCAACC	Δ*tfcP*
0363-F	GTCGTAGGGGGAGATTC	Δ*tfcP*
0363-G	CACGGATGAAGGCAAGC	Δ*tfcP*
0363-H	GACGGTTCATCCGCCTG	Δ*tfcP*
0361-0359-E	GCTCACCGGCTGGCGCCATG	Δ*fimU1/pilV1/pilW1*
0361-0359-F	CTTCGACCCGGCGAAGCACG	Δ*fimU1/pilV1/pilW1*
0361-0359-G	CATCGTCTTCAGTGACACGC	Δ*pilW1*
0361-0359-H	GGCGCGACAAGTTCATTGGG	Δ*pilW1*
0363 C91M C	**GCGCACCGGT**ATGGATACGCGC	*tfcP*^{C91M}
0363 C91H C	**GCGCACCGGT**CATGATACGCGCCTGC	*tfcP*^{C91H}
0363 C91X B	**GCGCACCGGT**CTTGGGTTTGATCTGG	*tfcP*$^{C91M/H}$
pilY1.1$^{D1173A+}$	CGAGACGGCAACTACGCCGTCATGTACGTGCCG	*pilY1.1*^{D1173A}
pilY1.1$^{D1173A-}$	CGGCACGTACATGACGGCGTAGTTGCCGTCTCG	*pilY1.1*^{D1173A}
0361-G_n	CCACCATGGCCATCCTGCTG	Δ*pilV1*
0361-H_n	CAGCTCAGGACGACGCGTAC	Δ*pilV1*
0359-G	CGGTGGCCATCGCCTCCATC	Δ*fimU1*
0359-H_n	GATGGCCTGGTTCTGGGTCG	Δ*fimU1*
0359 start XbaI	**GCGCTCTAGA**ATGAGGCACACACGCGGAATC	P_{pilA}-*fimU1*
0359 stop HindIII	**GCGCAAGCTT**TCAGTTCACGCACTCGATGGC	P_{pilA}-*fimU1*
0360 start XbaI	**GCGCTCTAGA**GTGAAGACGACTTTGACGC	P_{pilA}–*pilW1*
0360 stop HindIII	**GCGCAAGCTT**TCAATTTTCCGTCAGGAG	P_{pilA}–*pilW1*
0361 start XbaI	**GCGCTCTAGA**GTGAAGACGACTTTGACGCG	P_{pilA}-*pilV1*
0361 stop HindIII	**GCGCAAGCTT**TCACGGCGCCATCCTCGTC	P_{pilA}-*pilV1*
0362 start XbaI	**GCGCTCTAGA**GTGATGCGCACCCTCATCCAG	P_{pilA}-*pilY1.1*
0362 stop HindIII	**GCGCAAGCTT**TCACTGCGGCATCCTCCCGTC	P_{pilA}-*pilY1.1*
0364 start XbaI	**GCGCTCTAGA**GTGCAACGTCCCACAACC	P_{pilA}–*pilX1*
0364 stop HindIII	**GCGCAAGCTT**TCAAGGGGCGGGGGTGATG	P_{pilA}–*pilX1*
MalE Mfe	GCGC**CAATTG**ACCAACAAGGAC	Signal peptide MalE
MalE-SP rev	CTTGCCTTCATCCGTCGCGAGAGCCGAGGCGGAAA	Signal peptide MalE
0363_MalE-SP_fwd	GCCTCGGCTCTCGCGACGGATGAAGGCAAGCTCGC	TfcP untagged

	Oligonucleotides used for operon mapping	
**Name**	**Sequence**	**Combination**
0365 map-fwd	CCGAGCCATCCGAGGTG	A
pilX1 map-rev	GTGGGACGTTGCACCATGT	A
pilX1 q-1 fwd	AGGTCTCGACGATGACAATGG	B
tfcP q-1 rev	CCGCGGCCTTTGTTTTCTTC	B
tfcP q-2 fwd	ACAGAAGAAAACAAAGGCCGC	C
PilY1 map rev	CGGTGATTCCTCGGTGATG	C
PilY1 map fwd	CTGAGCCAGGACGAGAGCG	D
PilV map rev	AGCGTGATGTTCTCCATCGC	D
pilV1 q-2 fwd	CCCTCCATCCTCAGCACTATC	E
PilW map rev	TGAAGACGATGGGGGCATTC	E
pilW1 q-1 fwd	ATGTCGAACGTCTCTCGGTG	F
fimU1 q-1 rev	CATTCTCGCGTTGACGGTTG	F
	**Oligonucleotides used for qRT-PCR**	
**Name**	**Sequence**	**Gene**
fimU1_q-2_fwd	CTCCGGTGCGACAATGAATG	*fimU1*
fimU1_q-2_rev	TGTAGCAGAGCCCGTGAATC	*fimU1*
pilV1_q-1_fwd	GGACTGGATGAGAGCTACGTC	*pilV1*
pilV1_q-1_rev	GCTCGATAGTGCTGAGGATGG	*pilV1*
pilW1_q-1_fwd	ATGTCGAACGTCTCTCGGTG	*pilW1*
pilW1_q-1_rev	CGACAAGTTCATTGGGGTGC	*pilW1*
pilY1.1_q-2_fwd	GACGTCTCCCATTACGACCC	*pilY1.1*
pilY1.1_q-2_rev	AAGATGACTTCCTTCCCGCC	*pilY1.1*
rpsS_q-1_rev	GACGAACACCGGGATGAACT	*rpsS*
rpsS_q-2_fwd	GTTCGATCAAGAAGGGTCCGT	*rpsS*
tfcP_q-1_fwd	CCGACTCATCCTGTTGTCCC	*tfcP*
tfcP_q-1_rev	CCGCGGCCTTTGTTTTCTTC	*tfcP*
pilA_q-1_fwd	GATTCAACCCCCGCAACCG	*pilA*
pilA_q-1_rev	GTTCGTCTTCGCCTCGGAC	*pilA*
PilX1_q-6_fwd	GGTCGGAGGCTGGAACTC	*pilX1*
PilX1_q-6_rev	TCGTTTGGAGCGGGAAGG	*pilX1*
Tau_q-3_fwd	AGTGGAAGTCGTTGGTCTGC	*tuf2 (mxan_3298)*
Tau_q-3_rev	TTGGTGTGCGGGGTGATG	*tuf2 (mxan_3298)*

[1]Restriction sites are underlined. Oligonucleotide sequences that are not complementary to the template are indicated in bold.

Supplementary Table 4. Heavy labelled (^{13}C and ^{15}N) reference peptides with C-terminal Lys or Arg residue

Peptide Name	Peptide Sequence
FimU1_1	DFLDDLPALDAAAPGNLR
FimU1_2	IVVEENVPR
FimU1_3	SLIQVEPR
PilA_1	FGANSAIDDPTPVVAR
PilA_2	NAADLPVPAAGVPCISNDSFR
PilA_3	VSAAAGDCEVR
PilA_4	YSDFANEIGFAPER
PilB_1	ENLISVQQLR
PilB_2	HLVVPVNR
PilB_3	LGMSSLR
PilC_1	DILVFTR
PilC_2	KGEMEAMDVEAVNAR
PilC_3	TLGTMISSGVPILDALDVTAK
PilC_4	TVEDAIIYVR
PilM_1	DVTIGGNQFTEEIQK
PilM_2	QLNVSYEEAEALK
PilM_3	SLDFYAGTAADSNFSK
PilM_4	VLSSVAEQVAGEIQR
PilN_1	INLLPVR
PilN_2	LAVLDALR
PilN_3	MMDALASATPK
PilN_4	QSELEAHQAGVASTK
PilN_5	QVGGAQVGVPILVEFK
PilO_1	DIEELLAQINDIGKK
PilO_2	LSEALTELPEQR
PilO_3	VVLQSEFQATTFR
PilP_1	LVAVVTGDASPVAMVEDPAGR
PilP_2	QDPAYNMMTGR
PilQ_1	ALGKEEFGNIIR
PilQ_2	NIVVADDVSGK
PilQ_3	TNVLIVK
PilT_1	GASDLHVTTGSPPQLR
PilT_2	VHQIYSSMQVGQAK
PilV1_1	DLVPGVPDTAGNIANVR
PilW1_1	AGSGMGNAPIVFSDTR
PilW1_2	ALFEEQTMLAQVTGR
PilW1_3	INVVPGTGIETTTTDR
PilW1_4	LQPTTAPTTPALLVNPAR
PilW1_5	NLACHVEVTNVDAAGR
PilX1_1	QSPSGDAYAAFPLQTNVR
PilX1_2	YKEAYFAAEAGLAEGR
PilY1.1_1	SATVSGDLSPDIANDFVITK
PilY1.1_3	SSNIEHAFSTAK

PilY1.1_4	VNLDQVNPNAPLGQK
TfcP_1	AWLAGPNQIKPK
TfcP_2	GPSVDLGPVVPMR
TsaP_1	GDLVGPVGER
TsaP_2	IGVDLANSVPVTTQGFVTQR
TsaP_3	SLEELVPGDR
TsaP_4	YVVYHTTQAVK

Supplementary References

1. J. Sambrook, E. F. Fritsch, T. Maniatis, *Molecular Cloning: A Laboratory Manual* (Cold Spring Harbor Laboratory Press, 1989).

2. S. F. Altschul, W. Gish, W. Miller, E. W. Myers, D. J. Lipman, Basic local alignment search tool. *J. Mol. Biol.* **215**, 403-410 (1990).

3. F. Madeira *et al.*, The EMBL-EBI search and sequence analysis tools APIs in 2019. *Nucl. Acids Res.* **47**, W636-W641 (2019).

4. M. Blum *et al.*, The InterPro protein families and domains database: 20 years on. *Nucl. Acids Res.* **49**, D344-D354 (2021).

5. L. A. Kelley, S. Mezulis, C. M. Yates, M. N. Wass, M. J. Sternberg, The Phyre2 web portal for protein modeling, prediction and analysis. *Nat. Protoc.* **10**, 845-858 (2015).

6. J. Jumper *et al.*, Highly accurate protein structure prediction with AlphaFold. *Nature* **596**, 583-589 (2021).

7. M. Mirdita, S. Ovchinnikov, M. Steinegger, ColabFold - Making protein folding accessible to all. *bioRxiv* 10.1101/2021.08.15.456425, 2021.2008.2015.456425 (2021).

8. J. Yang, A. Roy, Y. Zhang, Protein-ligand binding site recognition using complementary binding-specific substructure comparison and sequence profile alignment. *Bioinformatics* **29**, 2588-2595 (2013).

9. W. Shi, D. R. Zusman, The two motility systems of *Myxococcus xanthus* show different selective advantages on various surfaces. *Proc Natl Acad Sci U S A* **90**, 3378-3382 (1993).

10. S. S. Wu, J. Wu, D. Kaiser, The *Myxococcus xanthus pilT* locus is required for social gliding motility although pili are still produced. *Mol. Microbiol.* **23**, 109-121 (1997).

11. I. Bulyha *et al.*, Regulation of the type IV pili molecular machine by dynamic localization of two motor proteins. *Mol. Microbiol.* **74**, 691-706 (2009).

12. A. Treuner-Lange *et al.*, PilY1 and minor pilins form a complex priming the type IVa pilus in *Myxococcus xanthus*. *Nat. Comm.* **11**, 5054 (2020).

13. S. Agrawal (2013) Identification and characterization of novel factors needed for two aspects of *Myxococcus xanthus* physiology: Social motility and osmoregulation. in *Bloomberg School of Public Health* (Johns Hopkins University), p 196.

14. B. Thomasson *et al.*, MglA, a small GTPase, interacts with a tyrosine kinase to control type IV pili-mediated motility and development of *Myxococcus xanthus*. *Mol. Microbiol.* **46**, 1399-1413 (2002).

15. S. Quan, A. Hiniker, J. F. Collet, J. C. Bardwell, Isolation of bacteria envelope proteins. *Methods Mol Biol* **966**, 359-366 (2013).

16. B. MacLean *et al.*, Skyline: an open source document editor for creating and analyzing targeted proteomics experiments. *Bioinformatics* **26**, 966-968 (2010).

17. F. W. Studier, Stable expression clones and auto-induction for protein production in *E. coli*. *Methods Mol. Biol.* **1091**, 17-32 (2014).

18. T. D. Schmittgen, K. J. Livak, Analyzing real-time PCR data by the comparative CT method. *Nat. Protoc.* **3**, 1101-1108 (2008).

19. J. Orans *et al.*, Crystal structure analysis reveals *Pseudomonas* PilY1 as an essential calcium-dependent regulator of bacterial surface motility. *Proc. Natl. Acad Sci. U S A* **107**, 1065-1070 (2010).

20. I. Bertini, G. Cavallaro, A. Rosato, Cytochrome c: occurrence and functions. *Chem Rev* **106**, 90-115 (2006).

21. C. Friedrich, I. Bulyha, L. Søgaard-Andersen, Outside-in assembly pathway of the type IV pilus system in *Myxococcus xanthus*. *J. Bacteriol.* **196**, 378-390 (2014).

22. B. Julien, A. D. Kaiser, A. Garza, Spatial control of cell differentiation in *Myxococcus xanthus*. *Proc Natl Acad Sci U S A* **97**, 9098-9103 (2000).

23. V. Jakovljevic, S. Leonardy, M. Hoppert, L. Søgaard-Andersen, PilB and PilT are ATPases acting antagonistically in type IV pilus function in *Myxococcus xanthus*. *J. Bacteriol.* **190**, 2411-2421 (2008).

24. M. Sun, M. Wartel, E. Cascales, J. W. Shaevitz, T. Mignot, Motor-driven intracellular transport powers bacterial gliding motility. *Proc. Natl. Acad Sci. U S A* **108**, 7559-7564 (2011).

25. E. Arslan, H. Schulz, R. Zufferey, P. Kunzler, L. Thöny-Meyer, Overproduction of the *Bradyrhizobium japonicum* c-type cytochrome subunits of the cbb3 oxidase in *Escherichia coli*. *Biochem Biophys Res Commun* **251**, 744-747 (1998).
